# Corticostriatal glutamate‐mediated dynamic therapeutic efficacy of electroacupuncture in a parkinsonian rat model

**DOI:** 10.1002/ctm2.70117

**Published:** 2024-12-03

**Authors:** Xinxin Jiang, Min Sun, Yitong Yan, Yanhua Wang, Xinyu Fan, Jing Wei, Ke Wang, Peirong Liang, Zirui Wang, Jihan Wang, Xiaomin Wang, Jun Jia

**Affiliations:** ^1^ Department of Physiology and Pathophysiology School of Basic Medical Science Capital Medical University Beijing China; ^2^ Beijing Tiantan Hospital Capital Medical University Beijing China; ^3^ School of Biomedical Engineering Capital Medical University Beijing China

**Keywords:** beta oscillatory synchrony, corticostriatal glutamate transmission, electroacupuncture stimulation, Parkinson's disease

## Abstract

**Background:**

Motor impairments are the defining cardinal features of Parkinson's disease (PD), resulting from malfunction of the cortico‐basal ganglia circuit. Clinical data have demonstrated that electroacupuncture (EA) stimulation may benefit motor symptoms in PD without adverse effects. However, the specific effects of EA on PD and the underlying mechanisms remain largely unclear.

**Methods:**

This study investigated the effects of EA stimulation during and after 100 Hz application in a rat model of PD created by unilateral injection of 6‐hydroxydopamine (6‐OHDA). To establish optimal treatment parameters of EA, motor behaviours were dynamically assessed using open field and rotarod tests. Additionally, we evaluated corticostriatal spine plasticity using immunoelectron microscopy and measured the levels of dopaminergic and glutamatergic neurotransmitters through microdialysis, in vivo electrochemistry and high‐performance liquid chromatography. Neural activity dynamics were recorded by measuring local field potentials in both the motor cortex and the striatum. Furthermore, chemogenetic techniques were employed to manipulate corticostriatal glutamatergic neurons and clarify the mechanisms that contribute to the therapeutic benefits of EA in the PD rat model.

**Results:**

Chronic EA stimulation resulted in a gradual and long‐lasting alleviation of motor symptoms, independent of nigrostriatal dopamine (DA) restoration. Notably, EA stimulation modulated corticostriatal spine plasticity and reduced excessive glutamate transmission in PD model rats. Moreover, EA effectively inhibited aberrant corticostriatal synchronised high‐beta (25–40 Hz) oscillations, which serves as a pathological biomarker of PD. Conversely, chronic chemogenetic activation of corticostriatal glutamatergic neurons hindered these positive outcomes of EA treatment in PD model rats.

**Conclusions:**

This study sheds light on the temporal dynamics and optimal parameters of EA treatment in PD. It emphasises the significance of inhibiting corticostriatal glutamate transmission in EA's therapeutic benefits for PD. Targeting glutamatergic neurons with EA holds promise as a non‐dopaminergic intervention for managing motor symptoms and abnormal neural activity with PD.

**Key points:**

- EA commonly protects dopaminergic neuronsby reducing neuroinflammation, oxidative stress, and apoptosis.
- New findings reveal that EA alleviates motor symptoms in a parkinsonian rat model without restoring striatal dopamine levels.
- EA effectively suppresses excessiveglutamate transmission and high‐beta synchronization, contributing to motorsymptom relief.
- Activation of corticostriatalglutamatergic projections may hinder the efficacy of EA.

## Graphical abstract

1. EA commonly protects dopaminergic neuronsby reducing neuroinflammation, oxidative stress, and apoptosis.
2. New findings reveal that EA alleviates motor symptoms in a parkinsonian rat model without restoring striatal dopamine levels.
3. EA effectively suppresses excessiveglutamate transmission and high‐beta synchronization, contributing to motorsymptom relief.
4. Activation of corticostriatalglutamatergic projections may hinder the efficacy of EA.

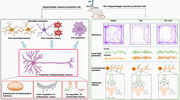

## 1 INTRODUCTION

Parkinson's disease (PD) is a common progressive condition marked by motor impairments stemming from the loss of dopaminergic (dopamine [DA]) neurons in the substantia nigra pars compacta (SNc), leading to nerve removal in the striatum.^1^ Although conventional drugs and surgical treatments are effective in treating PD, they have limitations and side effects.^2^, ^3^ As a result, there is growing interest in exploring novel approaches for PD management that offer therapeutic benefits without associated drawbacks.^4^

Acupuncture and/or electroacupuncture (EA) stimulation are non‐invasive alternative therapies for PD. Despite ongoing debates regarding their efficacy due to variations in study design and methodology across clinical trials,^5^, ^6^ reports indicate that acupuncture/EA stimulation may alleviate both motor and non‐motor symptoms in individuals diagnosed with PD.^7^, ^8^ Experimental studies in PD animal models, in which quantifiable parameters of EA stimulation such as intensity, frequency and duration are controlled, have also supported its beneficial effects.^9^, ^10^, ^11^, ^12^, ^13^ However, assessing the effectiveness of EA treatment is challenging due to the diverse range of stimulation parameters. Notably, investigations into the temporal dynamics of the effects of EA stimulation, including its sustained effects beyond the stimulation session,^14^ have been limited despite their crucial role in determining treatment efficacy.^15^

Accumulating evidence suggests that chronic high‐frequency EA stimulation effectively mitigates motor symptoms in PD models in the absence of restored nigrostriatal DA function.^9^, ^10^, ^11^, ^12^ This finding implies the engagement of a DA‐independent pathway in the therapeutic efficacy of EA. However, the specific mechanism responsible for the therapeutic effects remains unknown. Notably, the deterioration of DAergic neurons in SNc results in maladaptive alterations in the cortico‐basal ganglia circuit, leading to remodelling of glutamatergic synapses in striatal spiny projection neurons (SPNs) and elevated glutamate levels in the striatum.^16^, ^17^ This glutamate dysfunction significantly contributes to the motor deficits observed in PD.^18^, ^19^ Therefore, it is plausible that EA treatment ameliorates motor symptoms by normalising the elevated glutamate levels and restoring the balance of excitatory and inhibitory projections to the striatal SPNs, thereby re‐establishing the proper functioning of the cortico‐basal ganglia networks, even in the absence of restored nigrostriatal DA innervation.

Furthermore, corticostriatal glutamate transmission, the primary driving force of depolarisation in the striatum,^20^ regulates striatal neuronal activity and synchronised beta oscillations in PD.^18^, ^21^ Specifically, excessive beta oscillatory activity (13–30 Hz) in the cortico‐basal ganglia circuit has been recognised as a functional feature of PD and is correlated with the severity of parkinsonism and therapeutic responses.^22^ Conventional therapies for PD, such as levodopa and deep brain stimulation (DBS), effectively alleviate motor impairments and suppress excessive beta oscillations.^23^, ^24^ Hence, by modulating corticostriatal glutamatergic transmission, EA stimulation can help restore the abnormal beta oscillations, thereby mitigating the motor deficits in PD, even without restoring the nigrostriatal DA system.

The aim of this research was to examine the temporal dynamics of EA effects on motor impairments in a PD rat model and explore the involvement of glutamate in mediating the anti‐parkinsonian effects of EA. By investigating the temporal efficacy of EA stimulation on motor impairment and its impact on glutamatergic transmission and synchronised beta oscillations, our findings provide compelling evidence for a direct association between glutamate levels and the influence of EA stimulation on motor impairments. Chemogenetic inhibition of corticostriatal glutamatergic projections was shown to improve motor symptoms, while prolonged activation of the pathway diminished the efficacy of EA treatment. These findings offer invaluable insights for optimising treatment outcomes of EA and proposes targeting the corticostriatal glutamatergic pathway as an innovative and promising therapeutic strategy for PD.^25^

## 2 MATERIALS AND METHODS

### 2.1 Animals

Adult male Sprague‐Dawley rats, weighing 220–240 g, were procured from the Animal Centre at Capital Medical University in China and their use was approved by the institution. The rats were maintained in a regulated environment with a temperature of 22 ± 1°C and with a regular 12‐h light/dark cycle. They were ensured ad libitum access to food and water. Approximately 150 rats were utilised across the various experimental projects. Stringent measures were taken to minimise the number of rats used and to ensure their welfare, thereby reducing potential discomfort.

### 2.2 6‐OHDA lesion surgery protocol

The rats were anaesthetised with pentobarbital (40 mg/kg, i.p.) and securely positioned in a Kopf stereotaxic frame. Surgical procedures were performed by injecting 6‐hydroxydopamine (6‐OHDA; Sigma‐Aldrich) into the medial forebrain bundle (MFB) in the right hemisphere at the specified coordinates relative to bregma as follows: anteroposterior (AP) = −4.3 mm, mediolateral (ML) = −1.5 mm and dorsoventral (DV) = −7.6 mm. An infusion of 8 µg (5 µg/µL, 1.6 µL) of 6‐OHDA was delivered during the surgery. Sham rats underwent an identical surgical procedure but instead of receiving a saline injection. After a 2‐week recovery period, contralateral rotation behaviour was evaluated for 30 min after the subcutaneous injection of apomorphine (.05 mg/kg, Sigma‐Aldrich). Rats that exhibited at least five net contralateral rotations per minute were identified as models of PD.

### 2.3 EA stimulation

The experimental design is depicted in Figure S1A. Two weeks after lesions were induced with 6‐OHDA, the rats underwent daily EA stimulation for 30 min, 6 days a week, over a duration of 4 weeks. EA stimulation was applied to two specific acupoints included Dazhui (GV14), found below the spinous process of the vertebra prominens, and Baihui (GV20), located at the midpoint along the line connecting the tips of both ears on the parietal bone, as previously documented.^11^, ^12^ Briefly, two stainless steel needles measuring .25 mm in diameter and 5 mm in length were inserted obliquely at the designated acupoints. Stainless steel needles were connected to a Han's acupoint nerve stimulator (HANS), developed by the Neuroscience Research Institute at Peking University, to deliver bidirectional square‐wave electrical pulses with a duration of .2 ms. The stimulation intensity was escalated in a gradual manner, starting at 1 mA and progressing to 2 mA, and then further increased to 3 mA. Each increment was sustained for a period of 10 min. Control acupoints located approximately 3 mm from the tail in the gluteal muscle on each side were used, as previously reported.^10^

### 2.4 Behavioural assay

The rat's locomotor activity was evaluated through the open field test. Each rat was placed individually in a 50 × 50 × 50 cm cage, and their total movement distance and time of movement were recorded during a 30 min observation period. Motor coordination was evaluated by a rotarod instrument (Stoelting Company), measuring the time the rats remained on the rotating rod.

### 2.5 Histochemistry

Tyrosine hydroxylase (TH) immunostaining was conducted utilising a primary antibody (1:2000, Immuostar, Cat: 22941) and a biotinylated secondary antibody (Lab Vision). Stereological assessment was conducted with specialised software (Version 8.0, MBF Bioscience) to enumerate the number of TH‐positive cells in the SNc. The TH cell count was presented as a percentage relative to the unlesioned side. Additionally, Image‐Pro Plus software 6.0 was utilised to quantify the optical density of TH fibre immunoreactivity in the striatum. The TH fibre quantity is presented as a percentage relative to the unlesioned side.

### 2.6 In vivo microdialysis

Rats were anaesthetised with pentobarbital sodium for guide cannula implantation into the bilateral dorsolateral striatum. A precise hole was drilled at the following coordinates (AP: +.1 mm, ML: ± 2.6 mm), and a stainless steel guide cannula inserted subdurally (10.3 mm length, .86 mm outer diameter [OD]; CMA) was secured in place. After a 2‐week recovery period, a microdialysis probe with a molecular weight cutoff of 20 kDa (CMA) was implanted into the guide cannula. The rats were then placed in a free movement system, and artificial cerebrospinal fluid (ACSF) was continuously circulated through the probe at a flow rate of 2 µL/min until baseline values stabilised. Neurotransmitter content samples were collected at 20‐min intervals for subsequent analysis.

### 2.7 High‐performance liquid chromatography

Using a high‐performance liquid chromatography (HPLC) system (Model 5600A, CoulArray Detector System), all tissue samples (midbrain and striatum) and dialysate samples were examined. A modified HPLC method that incorporated electrochemical detection was employed to assess the concentrations of DA.^12^ For glutamate level measurements, the dialysate samples were combined with an equal quantity of o‐phthalaldehyde (Sigma) for pre‐column derivatisation. Isocratic elution was achieved using a mobile phase composed of 100 mM NaH_2_PO_4_ (pH 3.5), 10% methanol and .5 mM Na_2_EDTA.

### 2.8 In vivo electrochemistry

Recordings were conducted using the MT100 brain neurochemical analysis system (MT Technology Co., Ltd.) at the designated coordinates within the target region of the dorsal striatum: AP = +.1 mm, ML = ± 2.6 mm and DV = −4.0, −4.2, −4.5 and −4.7 mm. Carbon fibre microelectrodes with a diameter of 7 µm were electrochemically activated and used as working electrodes. An Ag/AgCl wire served as the reference electrode. The MT100 system acquired high‐speed voltammograms by rapidly applying a voltage ramp of 400 V/s across the range of −.4 to 1.3 V. This voltage ramp was repeated every 91.5 ms. KCl (70 mM) was locally applied for electrochemical recordings via a silica capillary tube attached to the carbon fibre electrode (CFE). Real‐time monitoring of the dynamics of DA content in the striatum was achieved by consecutive microinjections of 70 mM KCl at each recording site. Statistical analysis of DA levels was performed by converting the recorded current responses into DA concentrations (µM) using a linear equation.

### 2.9 Immunofluorescence

Coronal sections of rat brains were prepared with a depth of 40 µm. For immunofluorescence (IF), the sections were treated with the following primary antibodies: anti‐vesicular glutamate transporter type 1 (vGluT1; 1:1000, AB5905, Millipore); anti‐vesicular glutamate transporter type 2 (vGluT2; 1:2000, AB2251‐I, Millipore); anti‐αCaMKII (1:1000, SAB4503244, Sigma); or anti‐mCherry (1:2000, LS‐C204825, LSBio, Polyclonal Chicken Anti‐mCherry Antibody). Imaging was performed using a Zeiss 880 laser scanning confocal microscope (Zeiss).

### 2.10 vGluT1 and vGluT2 puncta detection and analysis

Serial images of vGluT1 and vGluT2 puncta were obtained from randomly selected subfields of the dorsolateral striatum. A total of at least five subfields per section from three to four sections per animal (four rats) were captured using a 63× objective with a 1.4 NA and a 5.4‐fold digital magnification (*x*/*y*, 1024 × 1024 pixels; 20 nm per pixel; *z* step, 400 nm). The regions of interest (ROIs) were determined by overlaying a randomly superimposed grid (frame size, 25 × 25 µm) on each image stack. Stereological counting was performed starting from an optical section located 2.0–3.0 µm below the surface of the slice and concluded in the same region. To enhance the signal‐to‐noise ratio, data from eight frames per image were averaged. The abundance of vGluT1‐ and vGluT2‐immunoreactive puncta was analysed using NIH ImageJ and quantified using the optical dissector method.^26^, ^27^

### 2.11 Golgi impregnation

Golgi staining was performed to impregnate striatal neurons using the Hito Golgi‐Cox OptimStain™ Kit (Hitobiotec). Rats were perfused with a fixation solution, and forebrain tissue was immersed in impregnation solution for 2 weeks. Coronal sections (120 µm) were stained, allowing for the visualisation and tracing of Golgi‐impregnated SPNs within the dorsolateral striatum. Neurolucida 360 software was used for analysis, including quantification of dendritic spine densities, measurement of morphological parameters (dendritic length and number of branches) and Sholl analysis of dendritic intersections.^27^

### 2.12 Immunoelectron microscopy

For immunoelectron microscopy (immuno‐EM), rats were perfused with a paraformaldehyde–glutaraldehyde solution. Tissue sections from the dorsolateral striatum were obtained and incubated with primary antibodies targeting vGluT1 or vGluT2. After rinsing, the sections were incubated with biotinylated secondary antibodies. Visualisation was achieved using 3,3'‐Diaminobenzidine (DAB), followed by post‐fixation with osmium tetroxide (catalogue no. 18456, PELCO) and staining with uranyl acetate. The sections were then dehydrated, embedded and subsequently sectioned. The ultrathin sections were prepared and mounted on grids, followed by staining and examination using a scanning electron microscope. The presence and localisation of vGluT1+ and vGluT2+ terminals were determined based on their immunoreactivity and the presence of clear vesicles. Spines were recognised by their diminutive size, connection to dendrites, distinct postsynaptic density (PSD) and/or the existence of the spine apparatus. Dendrites were recognised by their size, either oval or elongated shape, and the existence of microtubules and mitochondria. The density of synapses was determined by counting vGluT1+ or vGluT2+ immunolabelled terminals that made contact with spines or dendrites.

### 2.13 Implantation of microwire electrode arrays

Nichrome electrodes (33 µm; California Fine Wire Co.), coated with Formvar, were arranged in two 4 × 2 arrays at each site, with a spacing of 600 µm between the wires. Custom electrodes were fabricated for simultaneous unilateral insertion into the right motor cortex (AP = +1.5 mm, ML = −2.8 mm DV = −1.3 mm) and the ipsilateral dorsolateral striatum (AP = +.2 mm, ML = ‐2.8 mm DV = ‐3.8 mm). The wires were trimmed to match the required length for each specific implantation site of the array. Each electrode array contained 16 recording channels, accompanied by an additional electrode (∼1 mm in length with a scraped tip) serving as a local reference. Following the implantation, the animals were given a recovery period of 2 weeks.

### 2.14 Electrophysiological recordings

During recording sessions, the rats were positioned in an open field cage to collect electrophysiological data. A Cerebus acquisition system (Blackrock Microsystems) was used to record the signals with a filtering range of .1–5 kHz and a sampling rate of 30 kHz. The recorded signals were further filtered into distinct channels for offline analysis. This included local field potential (LFP) data from .1 to 250 Hz (downsampled to 1 kHz) and multiunit activity data from 250 to 5 kHz.

### 2.15 Single‐unit spike sorting

An Off‐line Sorter V3 (Plexon Inc.) was utilised for single‐unit spike sorting. Principal component analysis was used to isolate the spike waveforms into distinct clusters in three‐dimensional (3D) space. To be categorised as a single unit, a waveform needed to have a characteristic spike template and exhibit a sufficient refractory period (≥1 ms) between consecutive spikes. Objective measures, including the *F* statistic, J3 statistics and Davies–Bouldin validity index, were computed to assess the separation between the identified clusters within each recording channel.^28^, ^29^

### 2.16 Electrophysiological data analysis

The data analysis was conducted utilising Neuroexplorer (Blackrock Microsystems), Offline Sorter V3 (Plexon Inc.) and MATLAB (The MathWorks). The electrophysiological signals were divided into segments of 60 s. For every individual rat, the analysis utilised one segment for each behavioural state, incorporating data from a minimum of four electrodes in each brain region.

### 2.17 Power spectral analysis

Using Neuroexplorer, spectrograms were generated based on fast Fourier transform (FFT) to visualise the variations in spectral power throughout alert rest and walking epochs at different time points during the EA treatments. To calculate the total power spectral density within specific frequency bands, including alpha (8–12 Hz), low‐beta (12–18 and 19–25 Hz), high‐beta/low‐gamma (25–40 Hz) and high‐gamma frequencies (40–50 Hz), MATLAB scripts were utilised.

### 2.18 Coherence analysis

To assess the degree of coupling between the cortical and striatal LFPs, coherence analysis was performed. Using the following formula, the coherence *Cxy*(*f*) was computed for waveforms *x*(*t*) and *y*(*t*) at a particular frequency *f*:

$$Cxy\left(f\right)=\frac{{\left|P_{xy}\left(f\right)\right|}^{2}}{P_{xx}\left(f\right)P_{yy}\left(f\right)}$$

In this equation, *P_xy_* (*f*) indicates the cross‐power spectral density of the two signals, whereas *P_xx_* (*f*) and *P_yy_* (*f*) represent the power spectral densities of *x* and *y*, respectively. The pairwise correlation values were then averaged across the corresponding segments to obtain an overall measure of synchronisation.

### 2.19 Spike‐triggered waveform average

To investigate the relationship between spike trains and LFP oscillations, we analysed the distribution of instantaneous phases at the time of each spike to compute the spike–LFP phase vector. Phase locking between spike trains and LFP oscillations was assessed using the Rayleigh *Z* test, with 40 randomly selected spikes analysed per neuron. Neurons with fewer than 40 spikes were excluded from the analysis. A *p* value threshold of .05 was used to identify pyramidal neurons that exhibited significant phase locking to LFP oscillations. The strength of phase synchronisation was determined by the peak‐to‐trough magnitudes of spike‐triggered waveform averages (STWAs). To evaluate phase locking specifically with beta oscillations, spike trains were randomly shuffled 20 times, and the ratio of unshuffled to shuffled peak‐to‐trough magnitudes was calculated.

### 2.20 Chemogenetic virus delivery

A retrograde adeno‐associated serotype type 2 (AAV‐2) vector, controlled by the αCaMKII promoter (AAV2/retro‐αCaMKII‐P2A‐CRE‐WPRE‐pA, titre 2 × 10^12^ vg/mL) was performed unilaterally into the right striatum at the following coordinates (AP: +.2 mm, ML: −2.8 mm DV: −3.8 mm). Next, the ipsilateral motor cortex was infused at the coordinates (AP: +1.5 mm, ML: −2.8 mm DV: −2.0 mm) with either AAV2/9‐Ef1α‐DIO‐hM3D(Gq)‐mCherry‐WPRE‐pA (titre, 2 × 10^12^ vg/mL) or AAV2/9‐Ef1α‐DIO‐hM4D(Gi)‐mCherry‐WPRE‐pA (titre, 2 × 10^12^ vg/mL). These viruses selectively infected neurons expressing the Cre recombinase enzyme in the cortex, allowing the specific activation or inhibition of neurons projecting to the striatum from the motor cortex. Additionally, rAAV‐Ef1α‐DIO‐mCherry (titre, 2 × 10^12^ vg/mL) was used as a control virus and was infused into the motor cortex. All viruses were obtained from the BrainVTA Company. The injections were performed via an injection pump (Micro4, World Precision Instruments) with a 10 µL Hamilton syringe at a rate of 80 nL/min. The virus was allowed to express for a minimum of 4 weeks to ensure sufficient accumulation of the engineered proteins in the soma and axons. To selectively activate or inhibit infected neurons, we administered clozapine N‐oxide (CNO; Tocris) via i.p. injection (1, 3 or 10 mg/kg) or via drinking water (3 mg/kg/day) which was provided in lightproof containers.^30^

### 2.21 Statistical analysis

GraphPad Prism 8.0 (GraphPad Software) was utilised to conduct the statistical analysis. The normality of all datasets was evaluated using the Kolmogorov–Smirnov test. If the data exhibited a non‐normal distribution, non‐parametric tests were applied. In single‐factor experiments with two or more groups, we applied either a two‐tailed Student's *t*‐test or a one‐way analysis of variance (ANOVA), followed by a Bonferroni post hoc test. To compare the effects before and after CNO administration within the same animal, a two‐tailed paired‐sample *t*‐test was used. For experiments involving multiple factors, two‐way ANOVA with Bonferroni test was applied. Data are shown as means ± standard error of the means (SEMs), with statistical significance defined as *p* < .05.

## 3 RESULTS

### 3.1 Treatment with 100 Hz EA stimulation ameliorates motor deficits in 6‐OHDA‐lesioned rats in a time‐dependent manner

**FIGURE 1:**
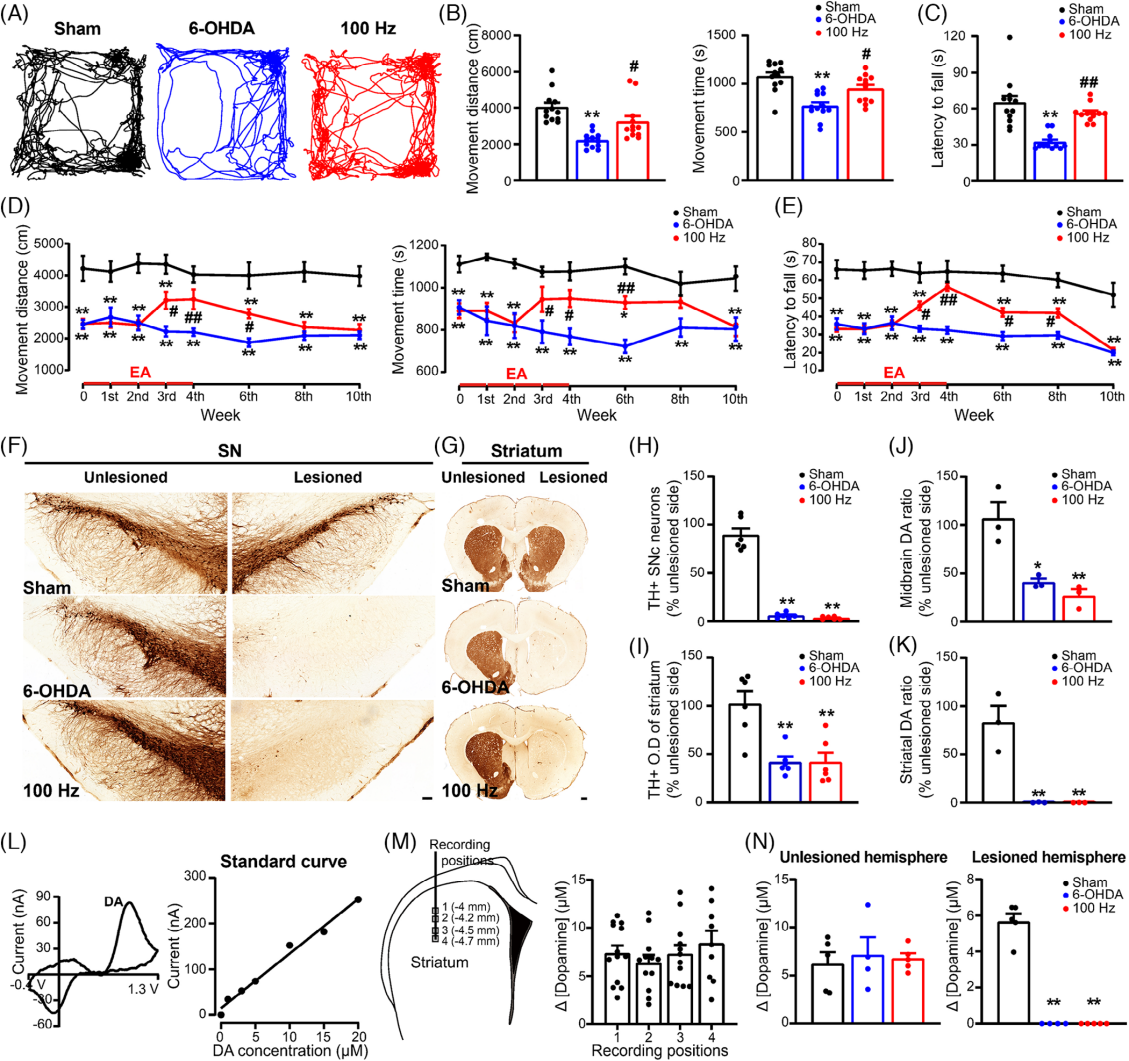
Electroacupuncture (EA) stimulation dynamically alleviates motor deficits in a parkinsonian rat model without rescuing nigrostriatal dopamine dysfunction. (A) Representative images demonstrating locomotor activity of sham‐operated, 6‐hydroxydopamine (6‐OHDA)‐lesioned and 6‐OHDA‐lesioned rats treated with 100 Hz EA. (B, C) Effects of 4 weeks of 100 Hz EA stimulation on the movement distance (left), movement time (right) and latency to fall (one‐way analysis of variance [ANOVA] with subsequent Bonferroni post‐test; movement distance, *F*(2, 33) = 14.19, *p* < .001; movement time, *F*(2, 33) = 14.32, *p* < .001; latency to fall, *F*(2, 33) = 19.89, *p* < .001; *n* = 12). (D, E) Temporal changes in movement distance, movement time and motor coordination following 100 Hz EA stimulation (two‐way ANOVA with a subsequent Bonferroni post hoc analysis; movement distance, *F*(2, 264) = 130.6, *p* < .001; movement time, *F*(2, 264) = 84.95, *p* < .001; latency to fall, *F*(2, 264) = 178.9, *p* < .001; *n* = 12). (F, G) Immunohistochemical labelling images of TH‐positive profiles in the substantia nigra pars compacta (SNc) and striatal terminals. Scale bars: 100 µm for the midbrain and 500 µm for the striatum. (H, I) Percentage of TH‐positive cells in the SNc and optical density of TH‐positive terminals in the striatum on the lesioned side (one‐way ANOVA followed by a Bonferroni correction test; neurons, *F*(2, 15) = 4.270, *p* = .034; terminals, *F*(2, 15) = 1.552, *p* < .001; *n* = 6). (J, K) Percentage of dopamine (DA) content in the midbrain and striatum on the side with lesions in relation to the side without lesions, as assessed through high‐performance liquid chromatography (HPLC) analysis (one‐way ANOVA with subsequent Bonferroni post‐test; midbrain, *F*(2, 6) = 15.62, *p* = .004; striatum, *F*(2, 6) = 22.82, *p* = .002; *n* = 3). (L) Representative cyclic voltammogram displaying the DA peak (left) and calibration of amperometric electrodes (right) in sham rats. (M) Schematic of electrode insertion (left) and graph (right) showing estimated DA release in response to KCl stimulation at different locations in the intact striatum (one‐way ANOVA with subsequent Bonferroni post‐test; *F*(3, 42) = .666, *p* = .578; position 1, *n* = 13; position 2, *n* = 12; position 3, *n* = 12; position 4, *n* = 9). (N) Bar graphs showing estimated DA release in response to KCl stimulation on the unaffected side (left) and the affected side (right) among the three groups (one‐way ANOVA with subsequent Bonferroni post‐test; unlesioned, *F*(2, 11) = .138, *p* = .873; lesioned, *F*(2, 11) = 137.4, *p* < .001; Sham, *n* = 5; 6‐OHDA, *n* = 4; 100 Hz, *n* = 5). Data from the different locations were averaged on each recording hemispheres. The data are presented as the means ± standard error of the means (SEMs). **p < .01, *p < .05 versus the sham group. ##*p* < .01, #*p* < .05 versus the 6‐OHDA group.

To examine the influence of EA on motor symptoms, we initially assessed motor impairments in PD rat model. After a 2‐week period, the rats with lesions induced by 6‐OHDA exhibited significant decreases in locomotion (Figure 1A), shorter distance travelled (Figure 1B) and impaired performance on the rotarod test (Figure 1C), indicating evident motor impairments. Next, we investigated the impact of EA stimulation at different frequencies on rats with 6‐OHDA‐lesions. Our findings showed that stimulation at 100 Hz led to notable improvements in motor deficits, while stimulation at 0 Hz, 2 Hz or 15 Hz had no discernible effect (Figure S1B,C). Additionally, we observed that 100 Hz EA stimulation applied at specific therapeutic acupoints (GV14 and GV20) produced significant positive effects on locomotion, distance travelled and latency to fall in the model rats, whereas stimulation of the control acupoints yielded no impact (Figure S1D,E). These findings provide compelling evidence that 4 weeks of 100 Hz EA at specific therapeutic acupoints effectively alleviates motor impairments in the 6‐OHDA‐lesioned rat model (Figure 1A–C).

Having identified the optimal parameters for EA stimulation, we explored the temporal dynamics of its effects in 6‐OHDA‐induced PD rats. During the first and second weeks of EA stimulation, no discernible alterations in motor symptoms were observed. However, in the third and fourth weeks of EA stimulation, we noted a significant increase in the distance travelled and movement time during the open field test, in addition to a prolonged latency to fall in the rotarod test (Figure 1D,E). Interestingly, these EA‐induced motor improvements persisted at week 6, which was 2 weeks after cessation of EA stimulation. However, by week 8, the improvements were no longer evident in the open field test, although they remained present in the rotarod test. Finally, at week 10, both tests indicated the loss of the therapeutic effects of EA stimulation (Figure 1D,E). These findings demonstrate that 100 Hz EA stimulation at specific acupoints produces a time‐dependent and enduring impact on motor behaviours in 6‐OHDA‐induced PD rats, with gradual improvements during the therapeutic period followed by a gradual waning of the beneficial effects over time.

### 3.2 EA stimulation does not restore nigrostriatal DAergic innervation in PD model rats

To investigate whether the favourable influence of EA stimulation on movement deficits is associated with the restoration of nigrostriatal DAergic function, we assessed SNc DAergic neurons and striatal DAergic innervation following 4 weeks of EA treatment. Immunohistochemical data showed that 6‐OHDA lesion resulted in a profound loss of TH‐positive cells (94.07 ± 1.16%) in the right SNc and a marked reduction in TH‐positive fibre innervation within the ipsilateral striatum. However, after 4 weeks of 100 Hz EA stimulation, there was no effect on the count of TH‐positive cells or the abundance of TH‐positive terminals (Figure 1F–I).

Subsequently, we examined DA levels in midbrain and striatum samples using HPLC. Comparing to the sham group, the group with 6‐OHDA‐induced lesions exhibited a significantly reduced midbrain DA content (Figure 1J) and almost complete depletion of DA in the striatum (Figure 1K). After 4 weeks of 100 Hz EA treatment, we did not observe any discernible impact on the reduction in DA content in the midbrain or striatum of rats subjected to 6‐OHDA lesions. These outcomes indicate that 4 weeks of 100 Hz EA treatment do not restore DA concentrations in the midbrain or striatum of the 6‐OHDA‐treated rats.

Furthermore, we assessed whether EA stimulation activates the residual nigrostriatal terminals to ameliorate motor impairments in the PD model rats. To examine this, we conducted in vivo chronoamperometric measurements and recorded cyclic voltammograms in the dorsal striatum to assess striatal DA release. Calibration of the carbon fibres from 1 µM to 20 µM exogenous DA indicated a linear response in the sham rats (Figure 1L). We noticed that KCl‐triggered DA release was consistent at various depths in the unlesioned striatum (Figure 1M). Conversely, the release of DA in the lesioned striatum of the PD model rats was significantly diminished compared to the intact striatum observed in the sham‐operated rats. Nevertheless, even after 4 weeks of 100 Hz EA stimulation, the maximal amount of DA released in the lesioned striatum remained low (Figure 1N), suggesting that EA treatment failed to reverse the severe impairment of DA release at nigrostriatal terminals. Collectively, our data suggest that the observed positive effects of EA treatment in ameliorating motor impairments are not linked to the restoration of tissue DA levels or a temporary increase in striatal DA release in rats with 6‐OHDA‐induced lesions.

### 3.3 Treatment with 100 Hz EA stimulation reverses the enhancement of corticostriatal spine plasticity and glutamatergic transmission in PD model rats

**FIGURE 2:**
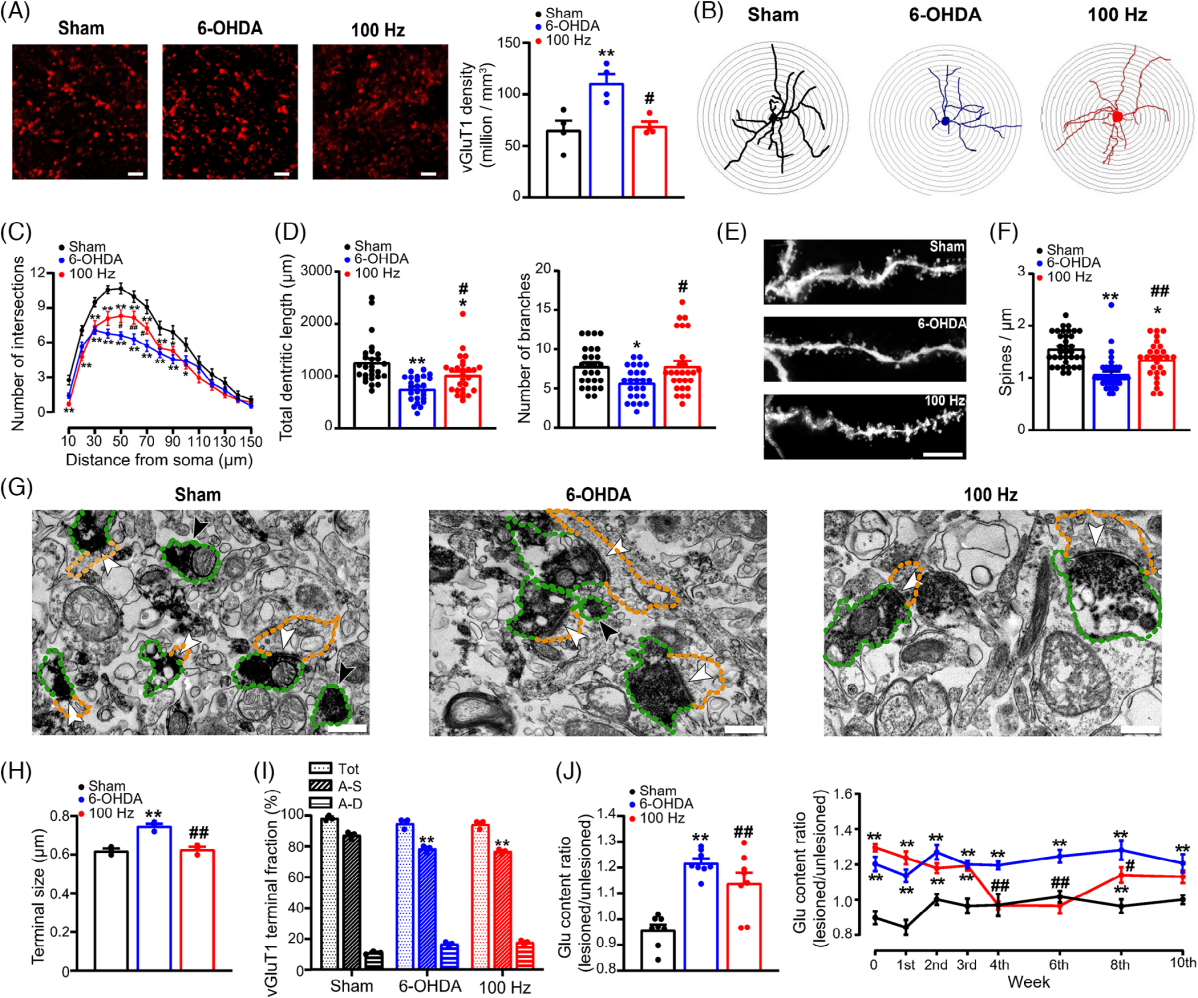
Electroacupuncture (EA) stimulation effectively regulates corticostriatal spine plasticity and mitigates aberrant glutamate transmission in a parkinsonian rat model. (A) Representative images (left) and population data (right) illustrating the density of corticostriatal axon terminals immunoreactive for anti‐vesicular glutamate transporter type 1 (vGluT1) in sham, 6‐hydroxydopamine (6‐OHDA)‐lesioned and 100‐Hz EA‐treated 6‐OHDA‐lesioned rats. Scale bar: 50 µm (one‐way analysis of variance [ANOVA] followed by Bonferroni's post‐test; *F*(2, 9) = 10.41, *p* = .005; *n* = 4). (B) Sholl plots illustrating changes in the dendrites of Golgi‐impregnated neurons. (C) A decreased number of dendritic intersections was observed in neurons from rats with 6‐OHDA‐induced lesions, but this reduction was reversed by 100 Hz EA stimulation (two‐way ANOVA with subsequent Bonferroni post‐test; *F*(2, 1140) = 89.62, *p* < .001; *n* = 25–27 neurons from six rats per group). (D) Total dendritic length (left) and number of branches (right) in Golgi‐impregnated neurons of untreated rats with 6‐OHDA lesions; these changes were ameliorated by 100 Hz EA stimulation (one‐way ANOVA with subsequent Bonferroni post‐test; length, *F*(2, 76) = 13.08, *p* < .001; branches, *F*(2, 76) = 4.847, *p* = .01; *n* = 25–27 neurons from six rats per group). (E) Representative images of dendritic spines on Golgi‐impregnated spiny projection neuron (SPN) dendritic segments. Scale bar = 10 µm. (F) Attenuation of spine loss on SPN dendritic segments with 100 Hz EA stimulation (one‐way ANOVA followed by a Bonferroni correction; *F*(2, 93) = 19.42, *p* < .001; *n* = 27–35 segments from six rats per group). (G) Electron microscopy images depicting vGluT1‐immunolabelled synaptic terminals (green outline) in the striatum, displaying asymmetric synaptic contacts with thick postsynaptic densities (PSDs; orange outline, white arrowhead) or vGluT1+ terminals (green outline) without synapse formation (black arrowhead) in the three groups. Scale bar: 500 nm. (H, I) Histograms of the size and number of vGluT1+ terminals (one‐way ANOVA with Bonferroni's post hoc test applied; size, *F*(2, 6) = 27.44, *p* = .001; A–S fractions, *F*(2, 6) = 26.94, *p* = .001; *n* = 3). (J) Changes in the glutamate content ratio in sham animals and on the lesioned and unlesioned sides of 6‐OHDA animals after 4 weeks of 100 Hz EA stimulation (left; one‐way ANOVA followed by the application of Bonferroni's post hoc test; *F*(2, 21) = 21.74, *p* < .001; *n* = 8). Time‐dependent alterations in the glutamate content ratio among the three groups (right; two‐way ANOVA with subsequent Bonferroni post‐test; *F*(2, 48) = 99.76, *p* < .001; *n* = 3). The data are presented as the means ± standard error of the means (SEMs). **p < .01, *p < .05 versus the sham group. ##*p* < .01, #*p* < .05 versus the 6‐OHDA group.

As the striatum receives not only nigrostriatal DAergic projections but also corticostriatal and thalamostriatal glutamatergic inputs,^31^ we next assessed the impact of EA treatment on glutamatergic projections following DA depletion. To achieve this goal, we first evaluated the density of vGluT1 and vGluT2, which serve as markers for cortical and thalamic glutamatergic axon terminals that project to the striatum. In the dorsolateral striatum, we noted an obvious increase in the density of vGluT1 puncta in sections from rats with lesions induced by 6‐OHDA, in comparison to those from sham‐treated rats (Figure 2A). However, following 4 weeks of EA treatment, the elevated abundance of vGluT1+ puncta in the striatum was attenuated. On the other hand, the number of vGluT2 puncta did not exhibit notable changes in the PD model group when compared to the sham group, and EA stimulation did not noticeably influence the density of vGluT2+ terminals (Figure S2A).

To further evaluate the postsynaptic alterations in Golgi‐impregnated SPNs in the dorsolateral striatum, we investigated spine density and dendritic arbour complexity. 3D morphometry analysis using Sholl analysis (Figure 2A) revealed fewer dendritic branches (Figure 2B) and shorter dendritic lengths (Figure 2C) in SPNs of rats with 6‐OHDA‐induced lesions compared to sham rats. After a 4‐week course of 100 Hz EA treatment, the pathological changes in dendritic branching and length were reversed (Figure 2D). We also observed a reduction in spine numbers in SPNs of 6‐OHDA‐lesioned rats; however, 4 weeks of EA stimulation significantly attenuated this reduction (Figure 2E,F).

Furthermore, we conducted immuno‐EM to investigate the ultrastructural features of vGluT1+ and vGluT2+ synapses. The majority of vGluT1+ and vGluT2+ terminals established asymmetric synapses characterised by larger PSDs (Figures 2G and S2B). In addition, we measured the size of vGluTs+ terminals, including those that did and did not contact spines or dendrites. The vGluT1+ terminals in the PD model group exhibited larger sizes compared to the sham group (Figure 2H). However, EA treatment resulted in smaller vGluT1+ terminals. Additionally, the number of axospinous terminals was diminished in the 6‐OHDA group, but EA treatment did not have a further impact on their numbers (Figure 2I). There were no notable differences observed in the size or quantity of vGluT2+ terminals among the three groups (Figure S2C,D).

Additionally, modifications in the striatal glutamate concentration during and after EA stimulation were detected through HPLC analysis of dialysate samples. The results demonstrated a significant rise in the striatal glutamate concentration in the PD model group compared to the sham group. And elevated glutamate levels persisted throughout the recording period following 6‐OHDA lesioning. However, after 4 weeks of EA treatment, we observed a gradual decrease in glutamate levels, and this reduction continued for 2 weeks after the discontinuation of EA stimulation. Importantly, the decreased glutamate levels were no longer sustained 4 weeks after the discontinuation of EA stimulation (Figure 2J). These findings suggest that EA dynamically modulates corticostriatal glutamatergic transmission, which is similar to the observed effects on motor behaviours. Electrophysiological tests were also conducted to evaluate glutamate‐mediated excitatory transmission in the striatum across three experimental groups. As illustrated in Figure S3, the analysis of spontaneous and minimal excitatory postsynaptic currents (sEPSCs and mEPSCs) in striatal spiny neurons revealed that EA treatment significantly reduced the 6‐OHDA‐induced increase in the frequencies of both sEPSCs and mEPSCs, while their amplitudes remained unchanged. This indicates that EA decreases facilitated corticostriatal glutamate release in PD rats. Collectively, these findings suggest that the renormalisation of glutamate‐mediated excitatory transmission in the striatum is associated with the reversal of motor deficits achieved through EA treatment.

### 3.4 Treatment with 100 Hz EA reduces corticostriatal high‐beta oscillatory synchronisation in PD model rats

Excessive synchronised beta oscillations have been recognised as a functional biomarker for parkinsonian motor symptoms.^22^ To further assess the dynamic therapeutic effects of EA stimulation, we conducted simultaneous recordings using long‐term implanted electrodes in the motor cortex and dorsolateral striatum. Our observations revealed an elevation in oscillatory LFPs activity, particularly within the high‐beta/low‐gamma frequency range of 25–40 Hz, in both nuclei 4 weeks after 6‐OHDA‐lesioned rats (Figure S4A–C). Elevated frequency‐specific power was observed during alert rest and walking epochs in the rats with 6‐OHDA‐induced lesions, with a greater increase in power observed during alert rest (Figure S4D–F). The peak frequencies of the dominant oscillations in the cortex and striatum were also elevated during walking, indicating a shift in the behavioural state of 6‐OHDA‐lesioned rats.^32^ Evaluation of LFP strength and coherence across the frequency range of 25–40 Hz focused on alert rest epochs to exclude movement‐related artefacts.

**FIGURE 3:**
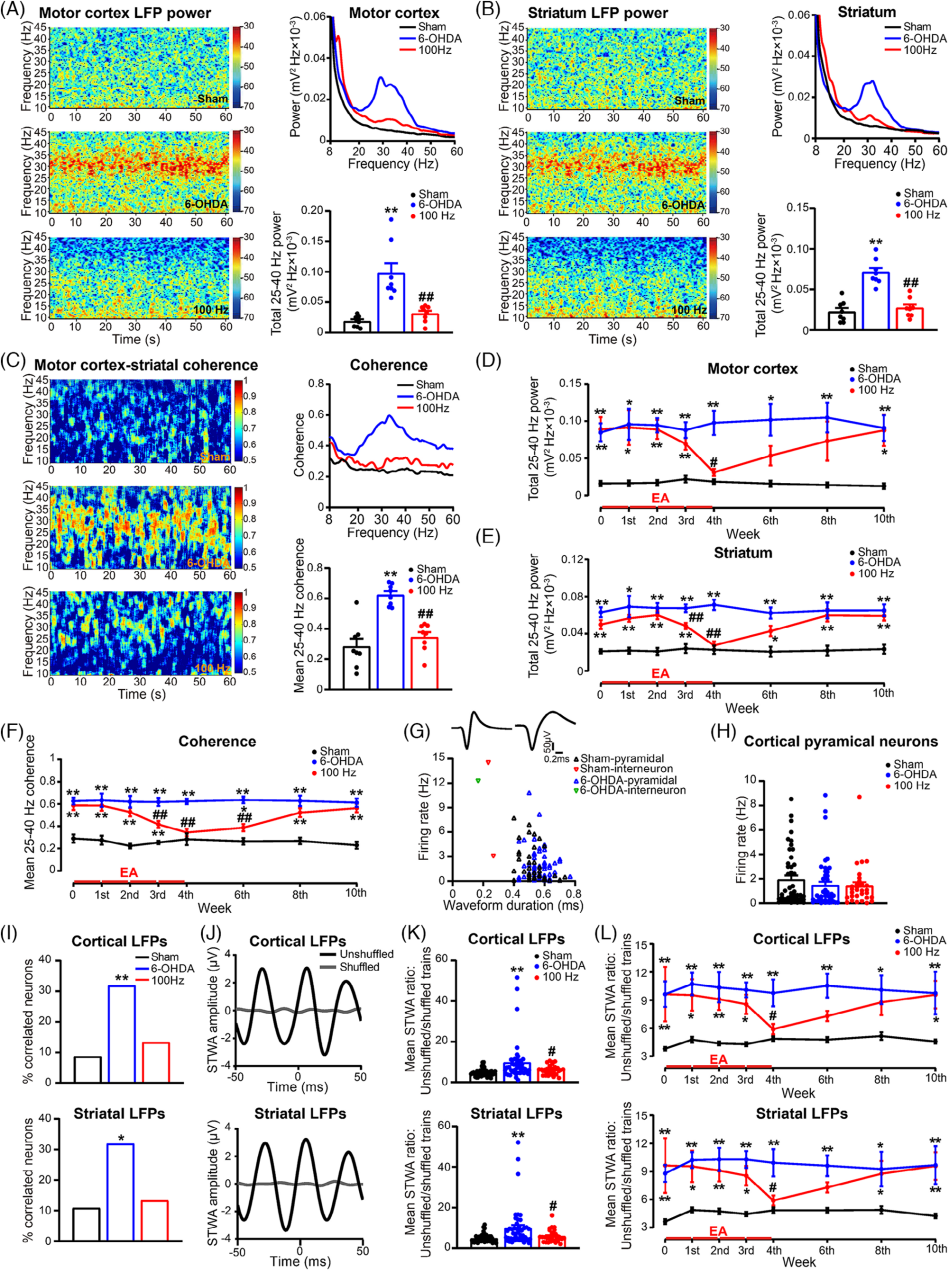
Electroacupuncture (EA) intervention dynamically modulates pathological corticostriatal beta oscillatory synchrony in a parkinsonian rat model. (A–C) Representative spectrograms and linear plots showing local field potential (LFP) spectral power and corticostriatal coherence in the motor cortex (A) and striatum (B) and corticostriatal coherence (C) during alert rest epochs (one‐way analysis of variance [ANOVA] followed by Bonferroni's post‐test; motor cortex, *F*(2, 21) = 18.13, *p* < .001; striatum, *F*(2, 21) = 30.76, *p* < .001; coherence, *F*(2, 21) = 22.60, *p* < .001; *n* = 8). (D–F) Time‐dependent effects of 100 Hz EA stimulation on LFP oscillatory activity and corticostriatal coherence in the lesioned hemisphere during alert rest epochs (two‐way ANOVA with subsequent Bonferroni post‐test; motor cortex, *F*(2, 168) = 66.14, *p* < .001; striatum, *F*(2, 168) = 132.2, *p* < .001; coherence, *F*(2, 168) = 189.6, *p* < .001, *n* = 8). (G) A scatter plot showing the waveform duration pattern (trough‐to‐peak) and firing rate of cortical neurons recorded in the sham and 6‐hydroxydopamine (6‐OHDA)‐lesioned rats. (H) Bar graphs demonstrating that 100 Hz EA stimulation had no significant impact on the firing rate of cortical pyramidal neurons (one‐way ANOVA with subsequent Bonferroni post‐test; *F*(2, 120) = .863, *p* = .424; sham, *n* = 46 neurons; 6‐OHDA, *n* = 47 neurons; 100 Hz, *n* = 30 neurons). (I) The percentage of cortical spike trains that were significantly phase‐locked to cortical (top) and striatal (bottom) 25–40 Hz LFPs among the three groups (chi‐square test; motor cortex, *χ^2^* (1) = 7.710, *p* = .006; striatum, *χ^2^* (1) = 6.100, *p* = .014). (J) Representative spike‐triggered waveform averages (STWAs) of pyramidal neurons for cortical LFPs (top) and striatal LFPs (bottom) in the beta band (25–40 Hz) in a 6‐OHDA‐lesioned rat. (K) Bar plots presenting the mean ratios of peak‐to‐trough magnitudes of unshuffled and shuffled STWAs for cortical LFPs (top) and striatal LFPs (bottom) within the 25–40 Hz frequency band for putative cortical pyramidal neurons (one‐way ANOVA with Bonferroni's post‐test; motor cortex, *F*(2, 120) = 7.471, *p* < .001; striatum, *F*(1, 120) = 7.807, *p* < .001; sham, *n* = 46 neurons; 6‐OHDA, *n* = 47 neurons; 100 Hz, *n* = 30 neurons). (L) Time‐dependent alterations in the mean STWA ratio between cortical pyramidal neurons and cortical (top) and striatal (bottom) 25–40 Hz beta oscillations among the three groups (two‐way ANOVA followed by Bonferroni's post‐test; motor cortex, *F*(2, 887) = 52.53, *p* < .001; striatum, *F*(2, 887) = 52.86, *p* < .001; *n* = 22–70 neurons). The data are presented as the means ± standard error of the means (SEMs). **p < .01, *p < .05 versus sham. ##*p* < .01, #*p* < .05 versus 6‐OHDA.

DA depletion resulted in a marked elevation in the magnitude of high‐beta oscillations observed in both the striatum and motor cortex (Figure 3A,B). Corticostriatal coherence also increased within the high‐beta range (Figure 3C). After 4 weeks of EA treatment, significant decreases were observed in the LFP power of the motor cortex and striatum, along with a reduction in corticostriatal coherence within the 25–40 Hz frequency band (Figure 3A–C). Furthermore, LFP power and corticostriatal coherence gradually decreased following EA treatment, with slightly smaller reductions in the cortex during the third week. The marked reduction in coherence induced by EA persisted for 2 weeks after termination of EA stimulation, although the reduction in LFP amplitude in the striatum and motor cortex was less evident during that period. The modulatory influence of EA on LFP activity gradually declined at 4 or 6 weeks following the cessation of EA stimulation (Figure 3D–F). In summary, EA treatment inhibits aberrant oscillations within the 25–40 Hz frequency range in these two brain areas, as well as corticostriatal coherence, in a time‐dependent manner.

Next, to investigate the correlation between cortical spiking and high‐beta oscillatory activity in these two brain regions, we calculated the STWA in both brain regions. Cortical neurons were categorised as presumed pyramidal neurons or interneurons (Figure 3G). The firing rate of pyramidal neurons did not significantly differ among the groups (Figure 3H). However, a greater percentage of cortical neurons exhibited phase‐locked to rhythms within the 25–40 Hz range in both the striatum and motor cortex of the PD model animals compared to sham rats (Figure 3I). EA treatment resulted in a reduction in the proportion of phase‐locked neurons, although the difference did not achieve statistical significance (Figure 3I). In addition, we monitored the mean peak‐to‐peak voltage of cortical single units over a period of 10 weeks (Figure S4I), which highlighted the reliability of the multichannel array in capturing stable firing dynamics.^28^

Furthermore, the averaged ratio of unshuffled to shuffled STWA peak‐to‐trough magnitudes within the 25–40 Hz frequency range was notably higher in PD model animals compared to sham animals (Figures 3J–L and S4G). After 4 weeks of EA stimulation, there was a significant reduction in the STWA ratio (Figure 3K). The decrease in the STWA ratio continued with EA treatment and this impact remained evident for a duration of 2 weeks after the discontinuation of EA stimulation (Figure 3L). These findings suggest that cortical spiking becomes synchronised with high‐beta LFP oscillations in PD and that EA stimulation dynamically modulates this abnormal spike–LFP phase locking. Overall, EA treatment has a dynamic modulatory effect on synchronised high‐beta oscillations in the corticostriatal pathway, similar to its beneficial effects on motor function, in PD model rats.

### 3.5 Selective corticostriatal inhibition alleviates beta oscillations, glutamate levels and motor impairments in PD model rats

**FIGURE 4:**
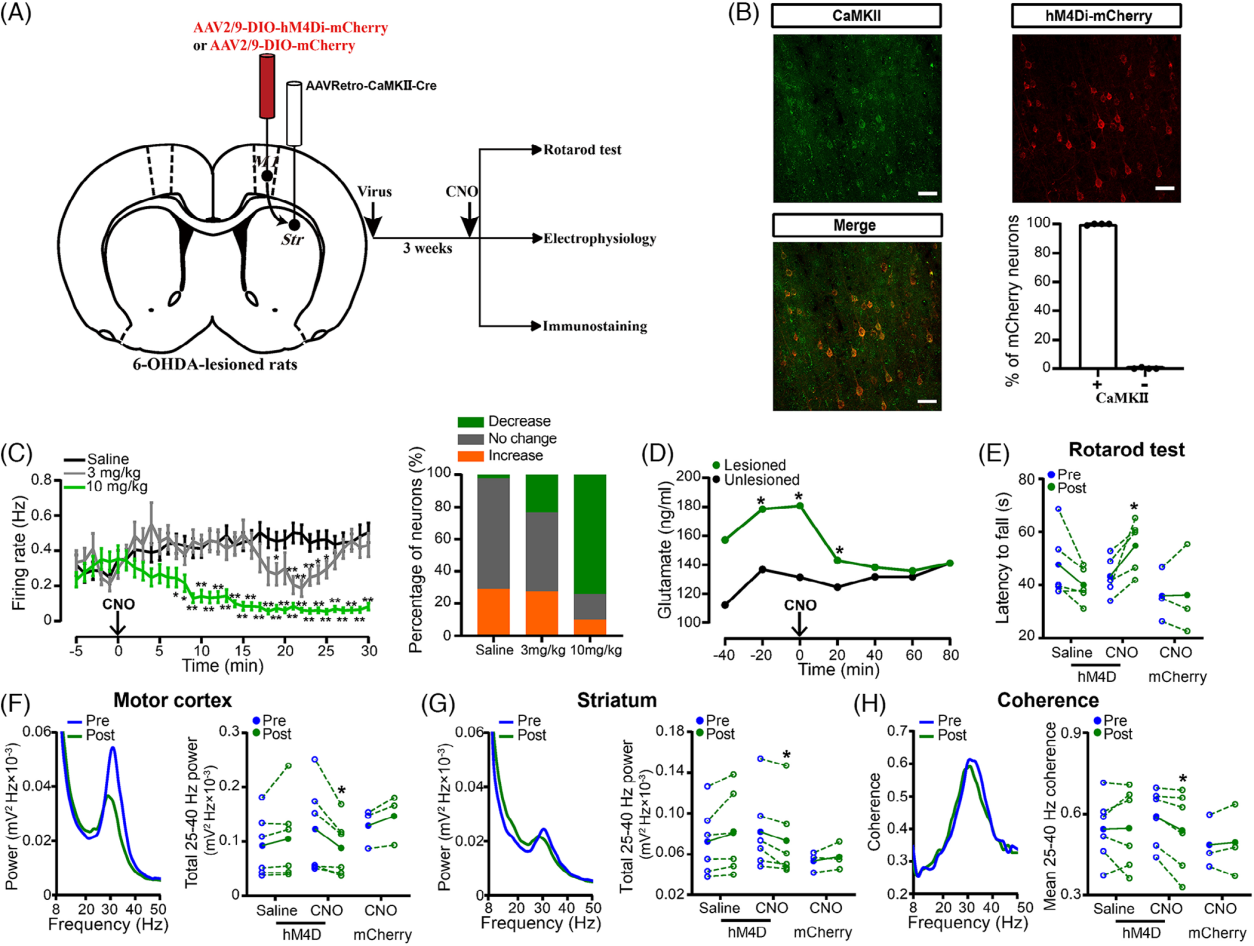
Inactivation of corticostriatal glutamatergic neurons alleviated motor symptoms and high‐beta oscillations in rats with 6‐hydroxydopamine (6‐OHDA) lesions. (A) Schematic illustrating the approach and timeline for viral vector injection. (B) Example images illustrating the expression of hM4Di‐mCherry in the motor cortex. Scale bar: 50 µm. Confirmation of the specificity of αCaMKII‐hM4Di DREADD for αCaMKII‐positive neurons and colocalisation analysis (*n* = 4 rats). (C) Changes in the discharge rate of putative cortical glutamatergic neurons in hM4Di‐expressing rats in response to saline or different doses of clozapine N‐oxide (CNO; 3 or 10 mg/kg, i.p., left; Repeated Measures [RM] two‐way analysis of variance [ANOVA] with Bonferroni's post‐test; *F*(2, 258) = 12.59, *p* < .001; saline, *n* = 86 neurons; 3 mg/kg, *n* = 94 neurons; 10 mg/kg, *n* = 81 neurons). **p < .01, *p < .05 versus saline. Statistical analysis of the number of putative cortical pyramidal neurons responsive to CNO or saline treatment (right). (D) Modifications in the levels of glutamate in the striatum on both the lesioned and unlesioned sides subsequent to CNO administration (20 min/bin, RM two‐way ANOVA with subsequent Bonferroni post‐test; *F*(1, 4) = 31.29, *p* = .005; *n* = 3). *p < .05 versus the unlesioned side. (E) Effects of chemogenetic inactivation of corticostriatal glutamatergic neurons on the latency to fall before and after CNO administration (10 mg/kg, i.p.) in hM4D‐CNO‐, hM4D‐saline‐ and mCherry‐CNO‐treated 6‐OHDA‐lesioned rats (paired two‐tailed *t*‐tests; hM4D‐CNO, *t*(4) = 3.084, *p* = .037; *n* = 5). (F–H) Changes in the average power spectra and synchronisation within the motor cortex (F) and striatum (G), as well as changes in total high‐beta power and mean high‐beta coherence (H) pre‐ and post‐CNO administration (10 mg/kg, i.p.) in hM4D‐saline‐, hM4D‐CNO‐ and mCherry‐CNO‐treated rats (paired two‐tailed *t*‐tests; hM4D‐CNO; motor cortex, *t*(5) = 2.616, *p* = .047; striatum, *t*(5) = 3.328, *p* = .021; coherence, *t*(5) = 2.893, *p* = .034; *n* = 6). *p < .05 versus preinjection. The data are presented as the means ± standard error of the means (SEMs).

If the motor impairments in PD caused by DA depletion are partially due to increased glutamatergic transmission in the corticostriatal pathway, inhibiting the activity of corticostriatal neurons may alleviate motor symptoms and desynchronise beta oscillations. To achieve selective inhibition of these neurons, we employed the inhibitory designer receptor exclusively activated by designer drugs (DREADD) hM4Di in 6‐OHDA‐lesioned rats (Figure 4A). Immunostaining for αCaMKII and hM4Di‐mCherry labelling were performed to validate cell specificity. Colocalisation analysis revealed that most mCherry+ neurons also expressed αCaMKII (Figure 4B), confirming that αCaMKII‐driven hM4Di expression was selectively confined to corticostriatal projection neurons.

Then, to assess the efficacy of hM4Di‐DREADD in modulating neuronal activity, we monitored spiking activity in the motor cortex. When rats expressing hM4Di were administered CNO in vivo (10 mg/kg, i.p.), a notable decrease in spike firing rates was observed in motor cortex neurons that express hM4Di, indicating the inhibitory effect of the hM4Di‐DREADD system (Figure 4C). CNO administration selectively inhibited the majority of neurons (60/81), while saline or lower doses (3 mg/kg) of CNO had no significant effect on neuronal firing (Figure 4C).

Moreover, microdialysis was performed to monitor the concentrations of glutamate in the dorsal striatum of both sides in rats expressing hM4Di and having 6‐OHDA‐induced lesions before and after the administration of CNO. The administration of CNO induced a significant reduction in glutamate levels, while glutamate levels on the unlesioned side remained stable (Figure 4D), indicating a decrease in corticostriatal glutamatergic transmission upon hM4Di activation.

Notably, CNO injection led to an increase in the latency to fall of hM4Di‐expressing rats with 6‐OHDA‐lesions, indicating a beneficial effect on motor function (Figure 4E). Additionally, CNO injection significantly reduced aberrant high‐beta oscillations in these two brain regions and desynchronised corticostriatal coherence (Figure 4F–H). No effects of CNO were observed on behaviour or electrophysiological activity in hM4Di‐CNO‐treated or mCherry‐CNO‐treated control rats (Figure S5), ruling out the possibility of off‐target effects. These results suggest that selective inhibition of corticostriatal glutamatergic neurons can alleviate motor symptoms and desynchronise abnormal corticostriatal beta oscillations in rats with 6‐OHDA‐lesions, supporting the role of the corticostriatal glutamatergic pathway in the pathological mechanism of PD.

### 3.6 The therapeutic effects of EA are reversed by chronic corticostriatal glutamatergic activation

**FIGURE 5:**
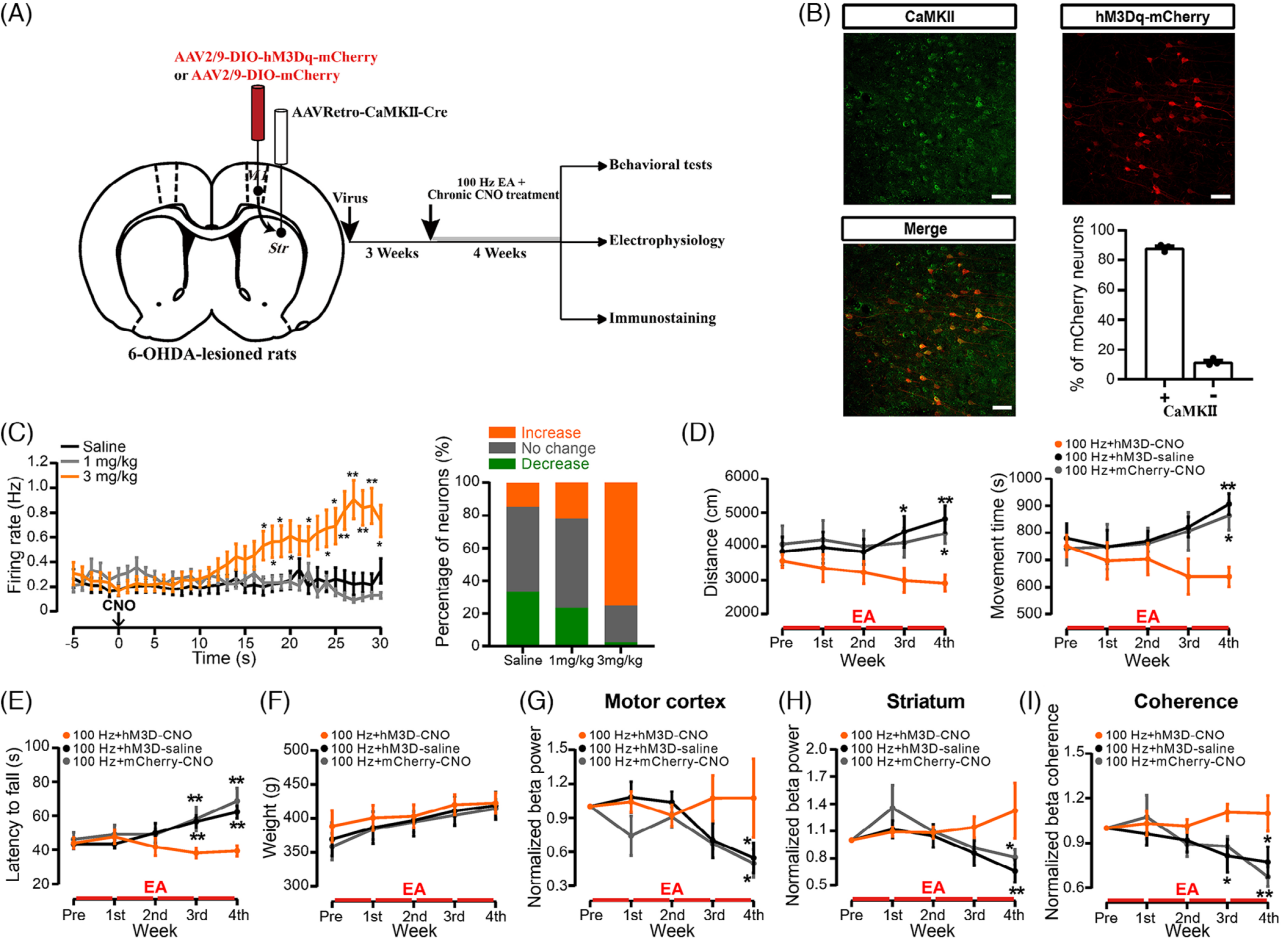
Reversal of electroacupuncture (EA) effects by chronic activation of corticostriatal neurons in rats with lesions caused by 6‐hydroxydopamine (6‐OHDA). (A) Schematic illustrating the setup and timeline of chronic clozapine N‐oxide (CNO) treatment (3 mg/kg/day) for 4 weeks, concurrent EA treatment and assessments at weekly intervals. (B) Representative images showing hM3Dq‐mCherry expression in the motor cortex. Verification of the cell‐type specificity of the αCaMKII‐hM3Dq DREADD in αCaMKII‐positive neurons, along with colocalisation analysis. Scale bar: 50 µm (*n* = 4 rats). (C) Left: Single administration of CNO (3 mg/kg, i.p.) induced greater activation of hM3Dq‐expressing cortical neurons compared to CNO (1 mg/kg, i.p.) or saline treatment (Repeated Measures [RM] two‐way analysis of variance [ANOVA] followed by Bonferroni's post‐test; *F*(2, 236) = 4.723, *p* = .0097; saline, *n* = 27 neurons; 1 mg/kg, *n* = 132 neurons; 3 mg/kg, *n* = 80 neurons). **p < .01, *p < .05 versus saline. Right: Statistical analysis of the number of putative cortical pyramidal neurons responsive to CNO or saline treatment. (D) Effects of hM3Dq‐DREADD‐mediated activation of corticostriatal neurons on locomotor activity in 6‐OHDA‐lesioned rats under chronic EA treatment. Distance travelled (left) and movement time (right) were measured for the 100 Hz + hM3D‐CNO, 100 Hz + mCherry‐CNO and 100 Hz + hM3D‐saline 6‐OHDA groups (RM two‐way ANOVA followed by a Bonferroni post hoc analysis; movement distance, *F*(2, 135) = 9.773, *p* < .001; movement time, *F*(2, 135) = 6.846, *p* = .002; 100 Hz‐hM3D‐CNO, *n* = 11; 100 Hz‐hM3D‐saline, *n* = 10; 100 Hz‐mCherry‐CNO, *n* = 9). **p < .01 versus 100 Hz + hM3D‐CNO. (E) Effect of hM3Dq‐DREADD‐mediated activation of corticostriatal neurons on the latency to fall in the rotarod test (RM two‐way ANOVA with subsequent Bonferroni post hoc testing; latency to fall, *F*(2, 125) = 10.62, *p* < .001; 100 Hz‐hM3D‐CNO, *n* = 11; 100 Hz‐hM3D‐saline, *n* = 9; 100 Hz‐mCherry‐CNO, *n* = 8). **p < .01 versus 100 Hz + hM3D‐CNO. (F) Effect of hM3Dq‐DREADD‐mediated activation of corticostriatal neurons on the weight of rats (RM two‐way ANOVA with subsequent Bonferroni post hoc testing; *F*(2, 135) = .881, *p* = .417; 100 Hz‐hM3D‐CNO, *n* = 11; 100 Hz‐hM3D‐saline, *n* = 10; 100 Hz‐mCherry‐CNO, *n* = 9). (G–I) Changes in power of high‐beta oscillations in the motor cortex (G) and striatum (H), as well as coherence (I), in rats with 6‐OHDA lesions that received treatment with 100 Hz + hM3D‐CNO, 100 Hz + mCherry‐CNO or 100 Hz + hM3D‐saline. The measurements were normalised to the power or coherence before EA treatment (RM two‐way ANOVA with a subsequent Bonferroni post hoc test; *F*(2, 135) = .881, *p* = .417; 100 Hz‐hM3D‐CNO, *n* = 11; 100 Hz‐hM3D‐saline, *n* = 10; 100 Hz‐mCherry‐CNO, *n* = 9). **p < .01, *p < .05 versus 100 Hz + hM3D‐CNO. The data are shown as the means ± standard error of the means (SEMs).

To further elucidate the contribution of corticostriatal glutamatergic transmission in the therapeutic effects of EA treatment in rats with 6‐OHDA‐lesions, we sought to counteract the effects of EA by activating hM3Dq‐DREADD through chronic administration of CNO. Figure 5A illustrates the schematic of the viral infusion along with the experimental timeline. Immunofluorescence staining revealed the selective expression of hM3Dq‐DREADD in cortical neurons, with 88.27 ± 1.26% of the hM3Dq‐expressing neurons expressing αCaMKII (Figure 5B). To test hM3Dq‐DREADD functionality, we recorded spiking activity in the M1 of virus‐injected 6‐OHDA rats. The application of a solitary dose of the DREADD agonist CNO (3 mg/kg, i.p.) to rats expressing hM3Dq significantly elevated the discharge rate of motor cortex neurons and led to a significant rise in the percentage of activated neurons (Figure 5C). However, neither saline nor 1 mg/kg CNO injection triggered the increased firing rate or the significant increased percent of activated neurons. These findings validate the hM3Dq‐DREADD system (3 mg/kg CNO) as an effective means to activation of corticostriatal glutamatergic projections.

After confirming hM3Dq‐DREADD robustly activates cortical neurons, we evaluated whether chronic corticostriatal projection neuron activation could hinder EA‐mediated improvements. As previously described protocol,^30^ we administrated CNO (3 mg/kg/day) in the drinking water of hM3Dq‐expressing rats with 6‐OHDA lesions for 4 weeks to induce continuous corticostriatal neuron activation, allowing us to assess its impact on the benefits of chronic EA treatment. As expected, EA treatment mitigated motor symptoms in rats with 6‐OHDA‐induced lesions expressing mCherry, even after 4 weeks of CNO administration. On the contrary, the chronic administration of CNO to rats expressing hM3Dq significantly dampened the effects of EA treatment on motor symptoms during the third and fourth weeks of the treatment period (Figure 5D,E). This indicates that selective chemogenetic activation of hM3Dq‐DREADD through chronic administration of CNO blocked the beneficial impact of EA on motor impairments in rats with 6‐OHDA‐induced lesions. Meanwhile, the weights of three groups of rats showed similar increasing trends during the 4‐week period. (Figure 5F).

Furthermore, LFP analysis unveiled that the application of EA treatment at 100 Hz in rats with 6‐OHDA lesions, both expressing mCherry following 4 weeks of CNO administration and expressing hM3Dq following saline administration, resulted in a gradual decrease in beta‐band power and coherence within the corticostriatal circuit (Figure 5G–I). This reduction became significant during the third week and remained prominent in the fourth week following EA treatment. However, in rats with 6‐OHDA lesions expressing hM3Dq and treated with CNO, the inhibitory effects of EA on beta frequency activity and synchronisation in the striatum and cortex were reversed (Figure 5G–I). Overall, these findings provide robust support for the hypothesis that the therapeutic effects of EA stimulation on movement deficits in rats with 6‐OHDA lesions rely on the attenuation of excessive corticostriatal glutamatergic transmission. However, sustained activation of hM3Dq‐DREADD with CNO effectively blocked the beneficial effects of EA treatment.

## 4 DISCUSSION

Acupuncture and EA are popular complementary therapies sought by PD patients due to their perceived benefits and minimal adverse effects.^5^ However, the therapeutic effects of acupuncture and the underlying biological mechanisms still remain elusive.^33^, ^34^ While recent studies have provided insights into the mechanisms of somato‐autonomic reflexes mediated by EA,^35^, ^36^ assessing the efficacy of EA treatment is challenging due to the influence of various stimulation parameters.^35^, ^37^, ^38^ In this study, our objective was to tackle this challenge by exploring the time‐dependent effects of EA stimulation and its impact on glutamatergic transmission and synchronised beta oscillations and by establishing a direct relationship between glutamate transmission and motor symptoms. This study demonstrated the temporal dynamics of the impact of EA stimulation on motor symptoms in rats with a PD model and revealed the involvement of glutamate in facilitating the antiparkinsonian effects of EA.

Interestingly, our study demonstrated that chronic EA stimulation resulted in a time‐dependent improvement in motor symptoms. However, it did not provide protection against degeneration of DAergic neurons in the severe parkinsonian model used in this study. This finding contradicts other reports suggesting that acupuncture and/or EA not only enhance motor activities but also rescue DAergic deficits in different rodent models of PD.^39^ This discrepancy may be attributed to variations in the severity of DA degeneration in different PD rodent models or the timing of EA intervention. Unlike the progressive decline of DAergic neurons observed in the 1‐methyl‐4‐phenyl‐1,2,3,6‐tetrahydropyridine (MPTP) model^13^, ^40^ or the partial 6‐OHDA lesion model,^9^, ^41^, ^42^ the irreversible neurodegeneration induced by 6‐OHDA lesioning in the MFB employed in our study may have limited the neuroprotective effects of EA stimulation.^43^ Additionally, EA stimulation at 2 or 4 weeks after 6‐OHDA injection may have been too late to promote endogenous neuroprotection.^44^ Despite the lack of neuroprotection, chronic EA stimulation still exerted beneficial effects on motor function, suggesting the involvement of other mechanisms, such as the modulation of corticostriatal synaptic plasticity.

The striatum receives glutamatergic innervation not only from the cortex but also from the thalamus. vGluT1 and vGluT2 are commonly used as specific markers to differentiate cortical from thalamic glutamatergic inputs to the striatum. Given the involvement of cortical glutamatergic inputs in spine pruning in the striatum in parkinsonism,^21^, ^45^, ^46^ it is reasonable to explore the consequences of chronic EA stimulation on modulating corticostriatal glutamatergic synaptic plasticity in a PD model. Consistent with previous studies,^21^, ^31^, ^46^, ^47^, ^48^ we noted a substantial decrease in the count of dendritic spines on individual SPNs and an increase in the overall number of vGluT1‐positive terminals in the striatum of rats with 6‐OHDA‐induced lesions. This seemingly paradoxical observation, where spine loss occurs alongside an increase in the number of synaptic inputs, is supported by morphological changes in asymmetric corticostriatal synapses, indicating increased glutamate transmission at the remaining synapses in the striatum due to DA denervation.^31^ Furthermore, we noted an enhancement in the size of presynaptic vGluT1+ terminals. Interestingly, previous studies have shown that the total number of thalamostriatal vGluT2+ synapses is not altered in the 6‐OHDA rodent model.^49^ However, DA depletion has been shown to selectively decrease thalamostriatal synaptic input onto direct pathway SPNs, potentially contributing to the reduced motor activity in PD.^50^ Consistent with these prior findings, in our present 6‐OHDA rat model, we did not observe evidence of changes in vGluT2‐positive thalamostriatal terminals. This differential response of corticostriatal and thalamostriatal glutamatergic inputs reflects structural and functional synaptic homeostatic adaptation occurs in SPNs in a parkinsonian state, which aligns with the notion of enhanced synaptic strength.^48^ This suggests that the loss of glutamatergic synapses primarily stems from the deafferentation of cortical inputs in PD models.^51^ Notably, chronic EA stimulation produced substantial effects on the presynaptic vGluT1+ puncta density, terminal size, postsynaptic spine number and complexity of dendrite arrangement in 6‐OHDA‐lesioned rats. However, we did not find evidence for a specific recovery of thalamostriatal synapses following EA treatment. Thus, chronic EA stimulation effectively modulates synaptic plasticity of corticostriatal glutamatergic projections in a rat model of PD.

Normalisation of excessive glutamate transmission could serve as a therapeutic strategy to normalise synaptic neurotransmitter levels.^25^ Therefore, our objective was to investigate the dynamic impact of EA stimulation on glutamate release, given its ability to modulate corticostriatal glutamatergic synapses. In this study, we observed that EA stimulation exerted a time‐dependent modulation of glutamate release within the striatum of PD rats. These findings suggest that EA stimulation gradually normalises excessive glutamate transmission, providing a crucial therapeutic approach to restore glutamatergic synaptic transmission to appropriate levels in rats with lesions induced by 6‐OHDA.^25^ Consequently, the modulation of glutamate release observed during and after EA stimulation is likely associated with its time‐dependent impact on motor symptoms in rats that have undergone 6‐OHDA‐induced lesions. In this study, we also found that EA treatment produces favourable motor outcomes in parkinsonian rats are associated with the normalisation of overactive glutamate transmission in the striatum. EA treatment significantly reduced the frequency of spontaneous and minimal glutamate‐mediated postsynaptic currents, while the amplitude remained unchanged. This suggests that EA applies an inhibitory effect on glutamate release from striatal glutamatergic terminals. Collectively, the morphological, biochemical and physiological data indicate that the reversal of corticostriatal glutamatergic transmission may underlie the therapeutic actions of EA in PD.

Furthermore, we investigated the impact of EA stimulation on corticostriatal glutamatergic neuronal activity, specifically focusing on aberrant beta oscillatory synchronisation, which serves as a potential biomarker for evaluating motor impairment and assessing therapeutic effects on PD‐related motor symptoms.^22^, ^52^, ^53^ Consistent with previous reports,^54^, ^55^, ^56^, ^57^ our findings revealed an increase in high‐beta/low‐gamma (25–40 Hz) oscillations in the DA‐depleted striatum and cortex of hemi‐parkinsonian rats. Significantly, EA stimulation effectively modulated synchronised corticostriatal beta oscillations during both the therapeutic and sustained phases, providing a pathophysiological basis for the application of time‐dependent interventions aimed at enhancing motor function in a PD rat model.

Building on the direct modulatory effects on corticostriatal neuronal activity, we further investigated the underlying mechanisms by utilising a retro‐DREADD approach to inhibit corticostriatal neurons.^41^, ^58^ Our study demonstrated that targeted chemogenetic inhibition of corticostriatal neurons effectively reduced synchronised corticostriatal beta oscillations, glutamate transmission and motor impairments in rats with 6‐OHDA‐induced lesions. This provides strong evidence supporting the essential role of heightened glutamate transmission in driving beta oscillations and motor signs in PD, indicating the therapeutic potential of inhibiting corticostriatal glutamate transmission. Furthermore, we explored the effects of water‐soluble CNO on the selective and sustained activation of the corticostriatal glutamatergic pathway.^30^ Surprisingly, this selective and sustained activation impeded the reduction in synchronised beta oscillations and the alleviation of abnormal motor behaviour mediated by EA stimulation in PD model rats. These findings collectively emphasise the significance of reducing excessive glutamate transmission in the corticostriatal circuit to achieve the positive effects of EA stimulation on motor impairments.

There are several limitations that warrant consideration. First, although we observed EA‐induced changes in corticostriatal synaptic plasticity and glutamate release in PD animals, further investigations are needed to establish if these changes lead to adaptive or time‐dependent enhancement in the expression or function of specific glutamate receptor subunits. Accordingly, we will assess changes in glutamate receptor subtypes following EA in the PD model, and future studies on related synaptic proteins may offer valuable insights. Thus, EA treatment that attenuates elevated glutamate transmission by decreasing its downstream signalling pathways or its release may offer a potential therapeutic approach. Moreover, the structural and functional connectivity of the corticostriatal network following EA treatment will be further investigated. This will reveal whether EA induces adaptive changes in the organisation and communication within this critical neural circuit.

Second, an intriguing aspect to explore is the potential impact of EA stimulation on the structural changes in corticostriatal circuitry. It would be valuable to investigate whether EA leads to a decrease in the quantity of vGluT1‐positive terminals while promoting the growth of postsynaptic spines and facilitating the formation of new synapses. While EA shows promise in reversing spine loss, additional studies are needed to comprehend the functional implications of these regrown spines. Uncovering the role and contribution of these spines in restoring glutamate levels and neuronal connectivity will offer valuable understanding the mechanisms that drive the therapeutic effects of EA in PD.

**FIGURE 6:**
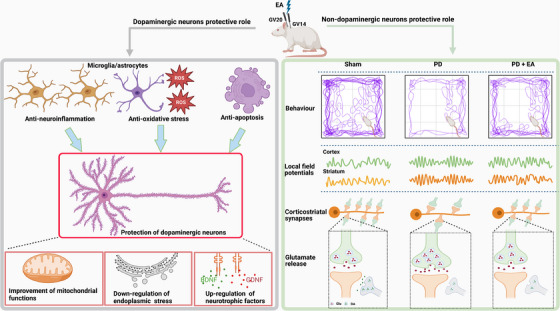
The left panel illustrates the neuroprotective effects of EA interventions on dopaminergic neurons through various mechanisms, including anti‐neuroinflammation, anti‐oxidative stress and anti‐apoptotic responses. These mechanisms involve enhancements in mitochondrial function, down‐regulation of endoplasmic reticulum stress and up‐regulation of neurotrophic factors, collectively promoting the survival of dopaminergic neurons. In contrast, the right panel presents findings demonstrating that EA alleviates motor deficits and synchronised beta oscillations by inhibiting excessive corticostriatal glutamate transmission in a PD rat model. Notably, these therapeutic effects occur without restoring the lost nigrostriatal dopamine system, emphasising a significant non‐dopaminergic protective role of EA interventions in PD. BDNF, brain‐derived neurotrophic factor; DA, dopamine; EA, electroacupuncture; GDNF, glial cell line‐derived neurotrophic factor; Glu, glutamate; PD, Parkinson's disease; ROS, reactive oxygen species.

Accumulating evidence indicates that EA stimulation provides neuroprotective effects on dopaminergic neurons through multiple approaches (Figure 6). These effects include anti‐neuroinflammation, anti‐oxidative stress and anti‐apoptotic responses, alongside modulation of the gut microbiome.^59^, ^60^ Furthermore, previous research indicates that EA can modulate glial function to exert anti‐inflammatory and antioxidant actions.^61^ Importantly, our present study highlights a significant non‐dopaminergic protective role of EA interventions in PD. EA stimulation alleviates motor deficits and synchronised beta oscillations by inhibiting excessive corticostriatal glutamate transmission in a PD rat model. Notably, these therapeutic effects occur without restoring the lost nigrostriatal dopamine system, demonstrating both neuroprotective and non‐neuroprotective roles of EA treatment in PD (Figure 6). Furthermore, the neuromodulatory effects of EA on astrocytes, which are crucial for maintaining glutamate homeostasis, necessitate further investigation into the mechanisms underlying synaptic plasticity and glial function modulation. Given these findings, we propose exploring the interactions between EA‐mediated changes in corticostriatal glutamate transmission (non‐neuroprotective role) and its effects on glial function (neuroprotective role). This exploration could provide critical insights into how EA mitigates the complex pathology of PD.

## 5 CONCLUSIONS

In summary, our findings highlight the time‐dependent and pathway‐specific effects of EA stimulation on motor impairments in a PD rat model. Importantly, the observed time‐dependent effect of EA treatment does not arise from the restoration of nigrostriatal DA innervation, but rather from its ability to modulate glutamate‐mediated corticostriatal synaptic plasticity and regulate excessive glutamatergic transmission in the PD rat model. Furthermore, we have discovered that the time‐dependent impact of EA stimulation on motor symptoms is intricately linked to its modulation of synchronised beta oscillations through changes in glutamate release. These discoveries offer novel perspectives on the regulatory mechanisms of the corticostriatal glutamatergic circuit and its relevance to the expression of motor symptoms in individuals with PD. Therefore, when designing EA treatment protocols for PD patients, it is crucial to consider the dynamic nature of the therapeutic response. Interventions that target specific time‐dependent alterations in glutamatergic signalling hold promise as innovative non‐dopaminergic therapies for PD.

## AUTHOR CONTRIBUTIONS

Xinxin Jiang and Min Sun prepared and performed most experiments. Yitong Yan, Yanhua Wang, Xinyu Fan and Peirong Liang performed a specific subset of the experiments and analyses. Jing Wei, Ke Wang, Zirui Wang and Jihan Wang contributed to the computational statistical analysis. Xiaomin Wang edited the paper. Jun Jia designed the experiments and prepared the paper. All authors reviewed and approved the final manuscript.

## CONFLICT OF INTEREST STATEMENT

The authors declare no conflicts of interest.

## ETHICS STATEMENT

All animal experiments conducted in this study were performed in compliance with a protocol reviewed and granted by the Animal Ethics Committee of Capital Medical University (AEEI‐2018‐055) and adhered to the protocols specified in the Guide for the Care and Use of Laboratory Animals published by the NIH. The reporting of animal studies in this research adheres to the ARRIVE guidelines.

## Supporting information



Supporting Information

## Data Availability

Not applicable.
