# Supplementary material for: Corticostriatal glutamate‐mediated dynamic therapeutic efficacy of electroacupuncture in a parkinsonian rat model
Source: Clin Transl Med. 2024 Dec 3;14(12):e70117. doi: 10.1002/ctm2.70117 (PMC11614550; doi:10.1002/ctm2.70117)
Supplement: Supplementary file 1 — Supporting Information [file CTM2-14-e70117-s001.docx]

**Supplementary Information for**

**Corticostriatal Glutamate-Mediated Dynamic Therapeutic Efficacy of Electroacupuncture in a Parkinsonian Rat Model**

Xinxin Jiang^1^, Min Sun^1,3^, Yitong Yan^1^, Yanhua Wang^1^, Xinyu Fan^1^, Jing Wei^2^, Ke Wang^1^, Peirong Liang^1^, Zirui Wang^1^, Jihan Wang^2^, Xiaomin Wang^1^, Jun Jia^1^*

Author Affiliations:

^1^ Department of physiology and pathophysiology, School of Basic Medical Science, Capital Medical University, Beijing 100069, China.

^2^ School of Biomedical Engineering, Capital Medical University, Beijing 100069, China.

^3^Beijing Tiantan Hospital, Capital Medical University, Beijing 100069, China.

Correspondence

*Jun Jia, Department of physiology and pathophysiology, School of Basic Medical Science, Capital Medical University, Beijing 100069, China.

E-mail: jiajun@ccmu.edu.cn

Xinxin Jiang and Min Sun contributed equally to this work.

FUNDING INFORMATION

This study was funded by the National Natural Science Foundation of China (No. 32271173 and No. 81774398) and the Natural Science Foundation of Beijing Municipality (No. 7242214).

**Supplemental information: Figure S1-5**

**Supplementary Figure S1**


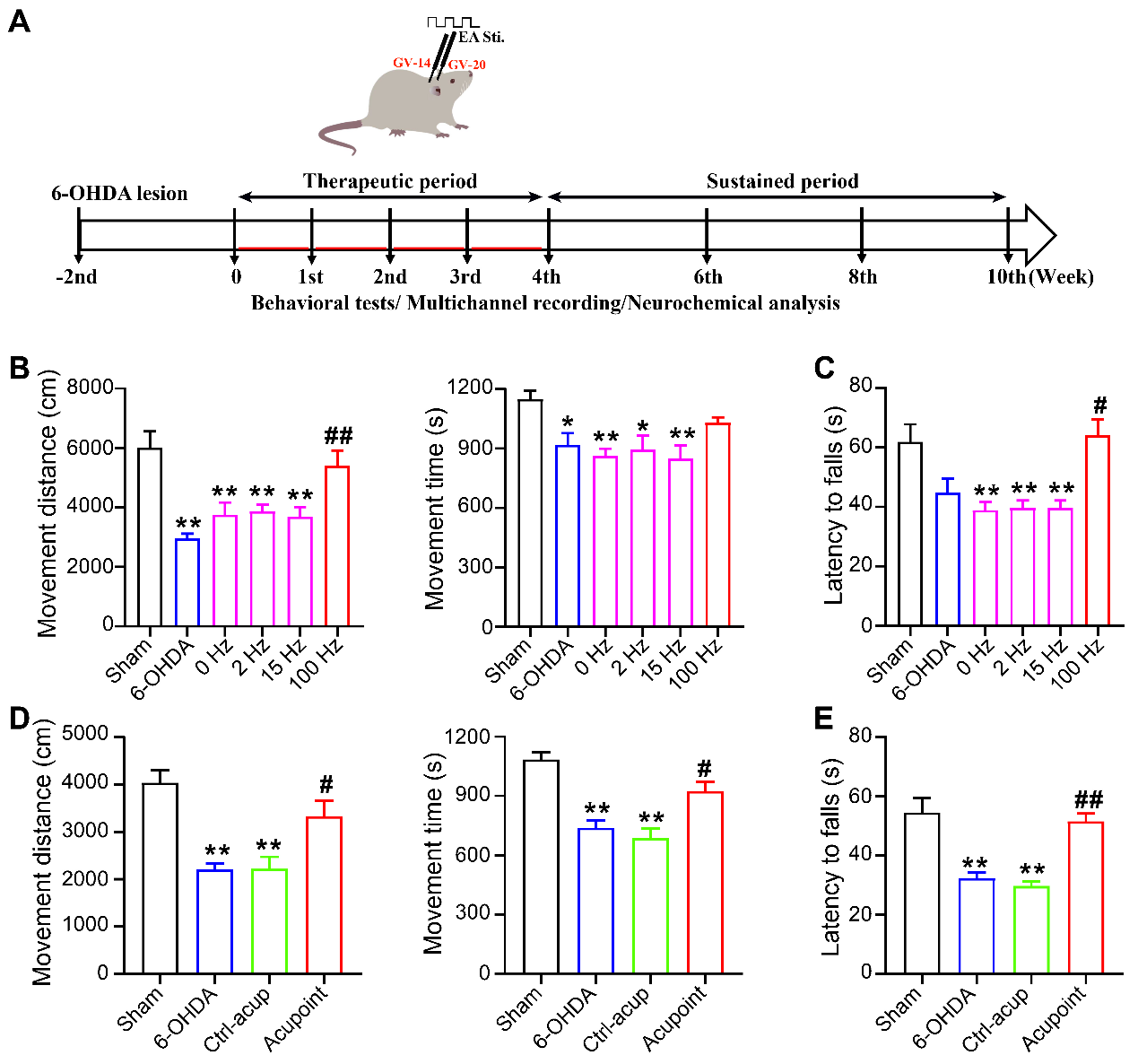


**Figure S1. Effects of EA stimulation at various frequencies and at either therapeutic or control acupoints in 6-OHDA-lesioned rats.** (A) Schematic illustration depicting the experimental timeline. EA stimulation was administered for four weeks during the therapeutic period (weeks 0-4), starting two weeks after 6-OHDA injection. The effects of EA were evaluated during both the therapeutic period and in the sustained period (weeks 6, 8 and 10) following the cessation of EA treatment. (B) Bar graphs illustrating the effects of four weeks of EA stimulation at different frequencies (0, 2, 15 and 100 Hz) on movement distance (left) and time (right) in the open field test (One-way ANOVA followed by Bonferroni’s post-test; movement distance, *F*(5, 66) = 9.006, *P* < 0.001; movement time, *F*(5, 66) = 4.934, *P* < 0.001; n = 12). (C) Bar graph illustrating the effect of four weeks of EA stimulation at different frequencies on the latency to fall in the rotarod test (One-way ANOVA followed by Bonferroni’s post-test; *F*(5, 66) = 7.872, *P* < 0.001; n = 12). Note that selective 100 Hz EA stimulation effectively reversed the reduced movement distance and latency to falls in the 6-OHDA lesioned rats. (D) Bar graphs illustrating the effects of four weeks of 100 Hz EA stimulation at the control acupoints and therapeutic acupoints on the movement distance (left) and time (right) in the open field test (One-way ANOVA followed by Bonferroni’s post-test; movement distance, *F*(3, 44) = 12.82, *P* < 0.001; movement time, *F*(3, 44) = 18.63, *P* < 0.001; n = 12). (E) Bar graph illustrating the effect of four weeks of selective 100 Hz EA stimulation at the control acupoints and therapeutic acupoints on the latency to fall in the rotarod test (One-way ANOVA followed by Bonferroni’s post-test; F(3, 44) = 17.17, P < 0.001; n = 12). Values are mean ± SEM. ***P* < 0.01, **P* < 0.05 vs. Sham. ##*P* < 0.01, #*P* < 0.05 vs. 6-OHDA.

**Supplementary Figure S2**


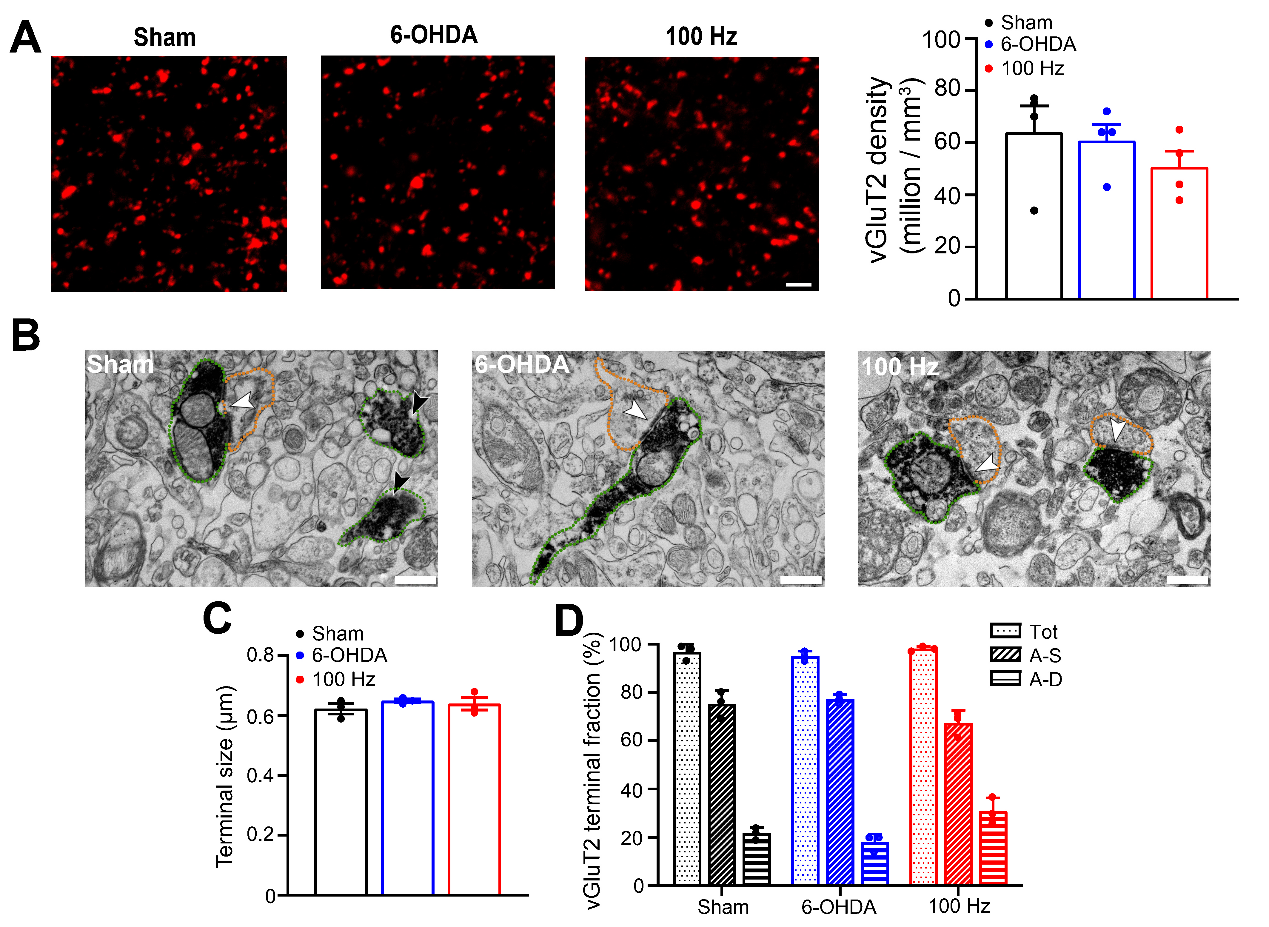


**Figure S2.** **Effects of 100 Hz EA on the thalamo-striatal projection and vGluT 2 immuno-EM.** (A) Representative images depicting the density of vGluT2-immunoreactive thalamostriatal axon terminals (depicted as red puncta) in sham, 6-OHDA, and 100-Hz EA-treated rats (top). Scale bar: 50 μm (One-way ANOVA followed by Bonferroni’s post-test; F(2, 9) = 0.807, P = 0.476; n = 4). The population data (bottom) illustrate that EA stimulation had no impact on the vGluT2 puncta density among the three groups. (B) EM images of vGluT2+ synaptic terminals showing asymmetric synaptic contacts, recognizable by a large PSD or vGluT2+ terminals without synapse formation in the rat striatum among the three groups. All images are at the same magnification. Scale bar: 500 nm. (C, D) Histogram comparisons of vGluT2+ terminal sizes (C) and terminal fractions (D) in the sham, 6-OHDA, and 100 Hz EA groups (One-way ANOVA followed by Bonferroni’s post-test; size, F(2, 6) = 0.656, P = 0.533; A-S fractions, F(2, 6) = 4.000, P = 0.079; n = 3). Note that there was no significant difference among the three groups.

**Supplementary Figure S3**

**Electrophysiology recording in brain slices**

In vitro electrophysiological recordings were conducted on brain slices from sham, 6-OHDA-lesioned, and EA-treated parkinsonian rats aged 14 to 16 weeks. After anesthetization, the animals were euthanized via cervical dislocation, and their brains were swiftly extracted. Coronal striatal slices, each 250 μm thick, were prepared using a vibratome (Leica, VT1200S, Germany) in a solution containing (in mM): 185 sucrose, 2.5 KCl, 1.25 NaH₂PO₄·2H₂O, 25 NaHCO₃, 25 glucose, 0.5 CaCl₂·2H₂O, and 10 MgSO₄. These slices were kept in artificial cerebrospinal fluid (ACSF) that was gassed with 95% O₂ and 5% CO₂ at 32 °C for 30 minutes, followed by an equilibration period of at least one hour at room temperature prior to recording. The ACSF composition (in mM) comprised 125 NaCl, 2.5 KCl, 1.25 NaH₂PO₄·2H₂O, 25 NaHCO₃, 10 glucose, 2 CaCl₂·2H₂O, and 1.5 MgSO₄. Single slices were then placed in a recording chamber, submerged in continuously flowing ACSF at 31 °C with a flow rate of 2.5–3 mL/min, and bubbled with a 95% O₂ and 5% CO₂ gas mixture.

Whole-cell patch-clamp recordings were carried out on striatal SPNs visualized using an Olympus microscope (BX51-WI, Olympus) equipped with a 40× long-working distance water objective (NA 0.8). Data acquisition and sampling were performed at 20 kHz, with filtering done using a low-pass filter set to 1 kHz (Axopatch 700B amplifier and Digidata 1440A, Molecular Devices). For the recording of spontaneous excitatory postsynaptic currents (sEPSCs), pipettes were filled with an internal solution consisting of (in mM): 140 K⁺-gluconate, 2 MgCl₂·6H₂O, 10 HEPES, 8 KCl, 2 Na₂ATP, 0.2 Na₂GTP, adjusted to pH 7.2 with KOH. To block GABA_A_ currents, bicuculline (10 µM) was added to the ACSF, and SPNs were clamped at a holding potential (Vh) of -70 mV for an 8-minute recording period. Subsequently, the bath solution was changed to include TTX (1 µM) and bicuculline (10 µM), allowing for the recording of spontaneous action potential-independent miniature excitatory postsynaptic currents (mEPSCs) for at least 8 minutes. The frequency and amplitude of sEPSCs and mEPSCs were analyzed using the Mini Analysis program (Synaptosoft), with a threshold set at 5 pA. Electrophysiological data are expressed as means ± SEM, with ‘n’ representing the number of recorded neurons.

**Figure S3**


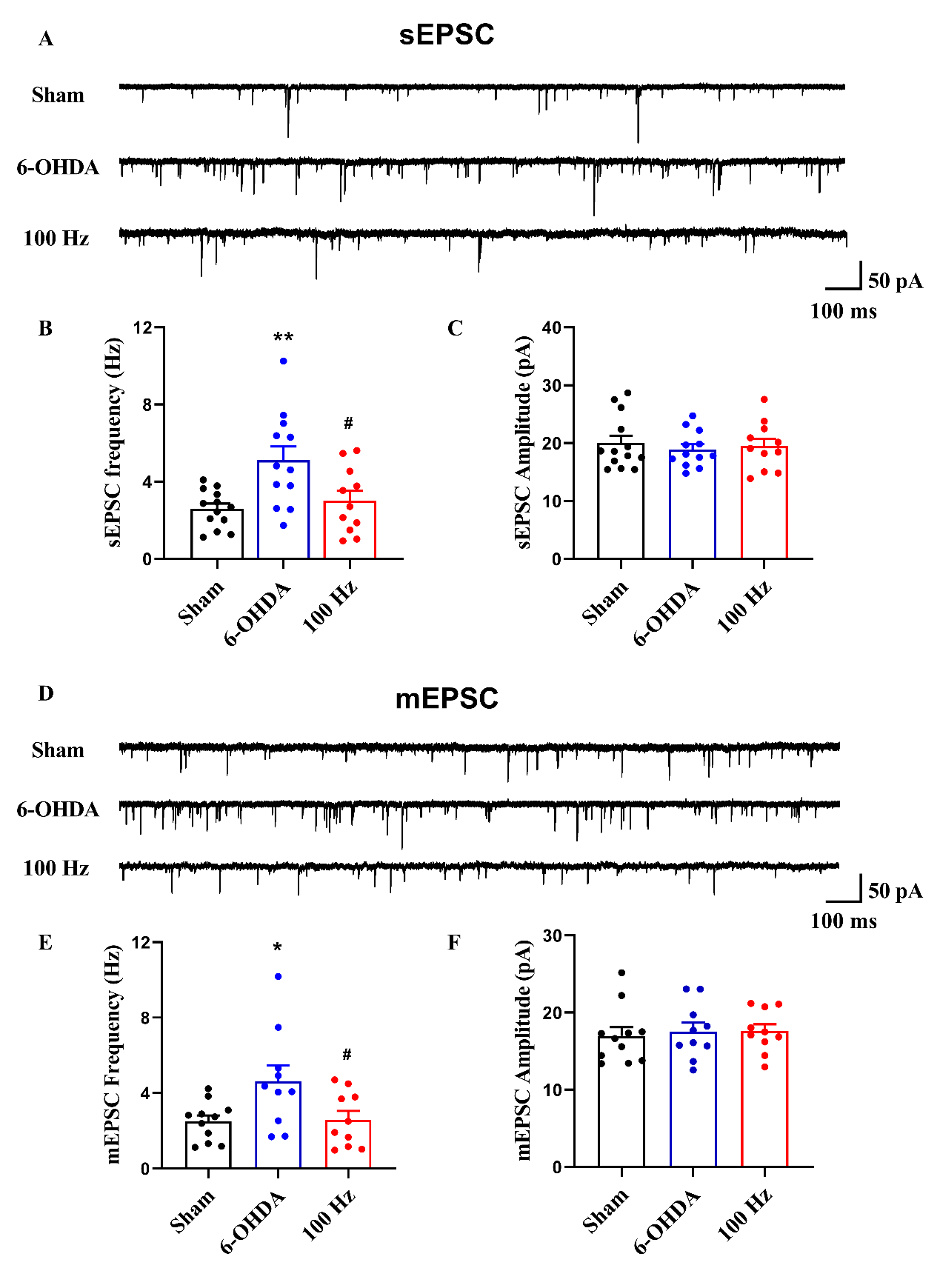


**Figure S3: Effects of EA treatment on the frequencies and amplitudes of sEPSCs and mEPSCs in striatal spiny neurons.**

(A) Representative traces of glutamatergic sEPSCs from sham, 6-OHDA-lesioned, and EA-treated parkinsonian rats. (B-C) Average sEPSC frequency (B) and amplitude (C) for each group, analyzed by one-way ANOVA followed by Tukey’s post-test (sEPSC frequency: *F*(2, 33) = 6.888, *P* < 0.01; sEPSC amplitude: *F*(2, 33) = 0.227, *P* > 0.05; sham: n = 13, 6-OHDA: n = 12, EA: n = 11). EA treatment significantly reduced sEPSC frequency in 6-OHDA-lesioned rats without affecting amplitude.

(D) Representative traces of glutamatergic mEPSCs across the three groups. (E-F) Average mEPSC frequency (E) and amplitude (F) for each group, analyzed by one-way ANOVA followed by Tukey’s post-test (mEPSC frequency: *F*(2, 28) = 4.514, P < 0.05; mEPSC amplitude: *F*(2, 28) = 0.113, P > 0.05; sham: n = 11, 6-OHDA: n = 10, EA: n = 10). EA treatment reduced mEPSC frequency in 6-OHDA-lesioned rats without affecting amplitude. Data are presented as means ± SEM. ***P* < 0.01, **P* < 0.05 vs. sham group; #*P* < 0.05 vs. 6-OHDA group.

**Supplementary Figure S4**


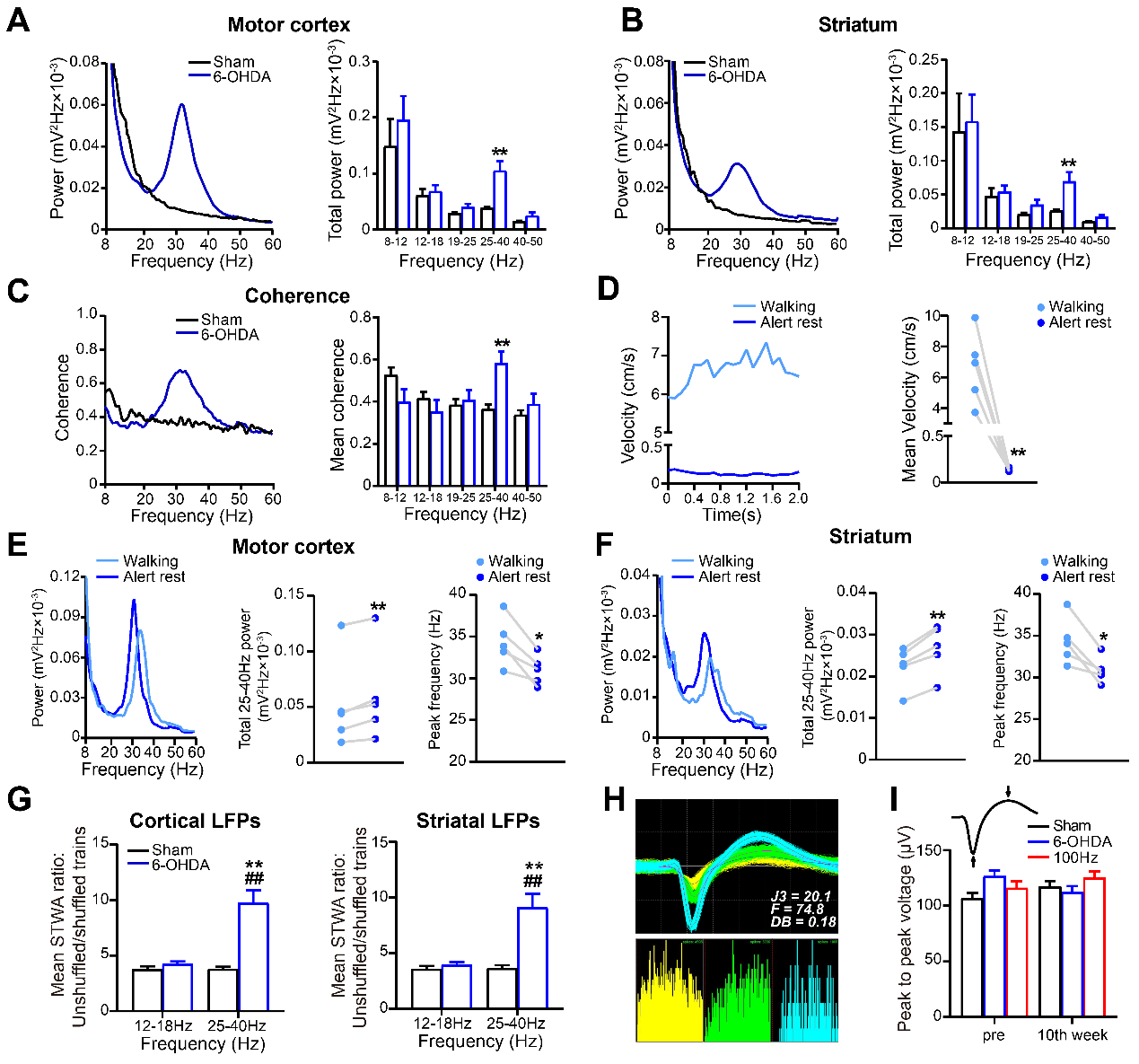


**Figure S4. Synchronization of high-beta oscillations in the motor cortex-striatal pathway in 6-OHDA-lesioned rats.** (A-C) Left: Linear graphs depicting the averaged LFP power spectra in the motor cortex (A) and striatum (B) as well as corticostriatal coherence (C) ranging from 8 to 60 Hz in sham rats (black) and 6-OHDA-lesioned rats (blue) two weeks after microwire positioning. Right: Bar graphs showing total LFP power in the motor cortex (A) and striatum (B) as well as corticostriatal coherence (C) across a series of frequency ranges, i.e., alpha (8-12 Hz), low beta (12-18 Hz and 19-25 Hz), high beta/low gamma (25-40 Hz), and gamma (40-50 Hz), in sham rats and 6-OHDA-lesioned rats (Unpaired two-tailed t-tests; motor cortex, t(22) = 3.842, P = 0.001; striatum, t(22) = 3.208, P = 0.004; coherence, t(22) = 3.703, *P* = 0.001; n = 12). ***P* < 0.01 vs. Sham.  (D) Left: Linear graph depicting the average movement velocity of 6-OHDA rats during alert rest and walking epochs. Right: Changes in the mean movement velocity of 6-OHDA-lesioned rats from walking epochs to alert rest epochs. Note the mean velocity of rat during walking is higher than that in alert rest epochs (Paired two-tailed t-tests; *t*(4) = 6.248, *P* = 0.003; n = 5). ***P* < 0.01 vs. walking epochs. **(E, F)** Left: Linear graphs depicting the average power spectra ranging from 8 to 60 Hz in the motor cortex (E) and striatum (F) during alert rest and walking epochs in 6-OHDA-lesioned rats. Middle: Changes in total 25-40 Hz LFP power in the motor cortex (E) and striatum (F) from alerts rest to walking epochs in 6-OHDA-lesioned rats (E: paired two-tailed t-tests; t(4) = 4.679, P = 0.0095; F: paired two-tailed t-tests; t(4) = 7.058, P = 0.002; n = 5 rats). Right: Changes in the peak frequency in the motor cortex (E) and striatum (F) from alert rest to walking epochs in 6-OHDA-lesioned rats (E; paired two-tailed t-tests; t(4) = 4.307, p = 0.013; n = 5 rats; F: paired two-tailed t-tests; t(4) = 4.045, P = 0.016; n = 5 rats). **P < 0.01, *P < 0.05 vs. walking epochs. (G) Bar graphs depicting the mean ratios between original STWAs and the mean of shuffled STWAs for LFPs in the frequency ranges of 12-18 Hz and 25-40 Hz for putative cortical pyramidal neurons (Two-way ANOVA followed by Bonferroni’s post-test; cortical LFPs, *F*(1, 198) = 14.08, *P* < 0.001; striatal LFPs, *F*(1, 198) = 10.37, *P* = 0.002; Sham, n = 39 neurons; 6-OHDA, n = 62 neurons). ***P* < 0.01 vs. Sham. ##*P* < 0.01 vs. 12-18 Hz. (H) Examples of spike cluster separation. Upper: Identified clusters in 3D PC space. Lower: Waveforms of neurons recorded in the same channel. The F, J3 and DB statistics were calculated and are shown at the bottom right. (I) The distribution of peak-to-peak voltage in the sham, 6-OHDA-lesioned and 100 Hz EA-treated rats before and after the 10th week of recording. The inset is an example of the peak-to-peak voltage of a single unit (Two-way ANOVA followed by Bonferroni’s post-test; *F*(2, 184) = 1.501, *P* = 0.226; n = 23-39 neurons).

**Supplementary Figure S5**


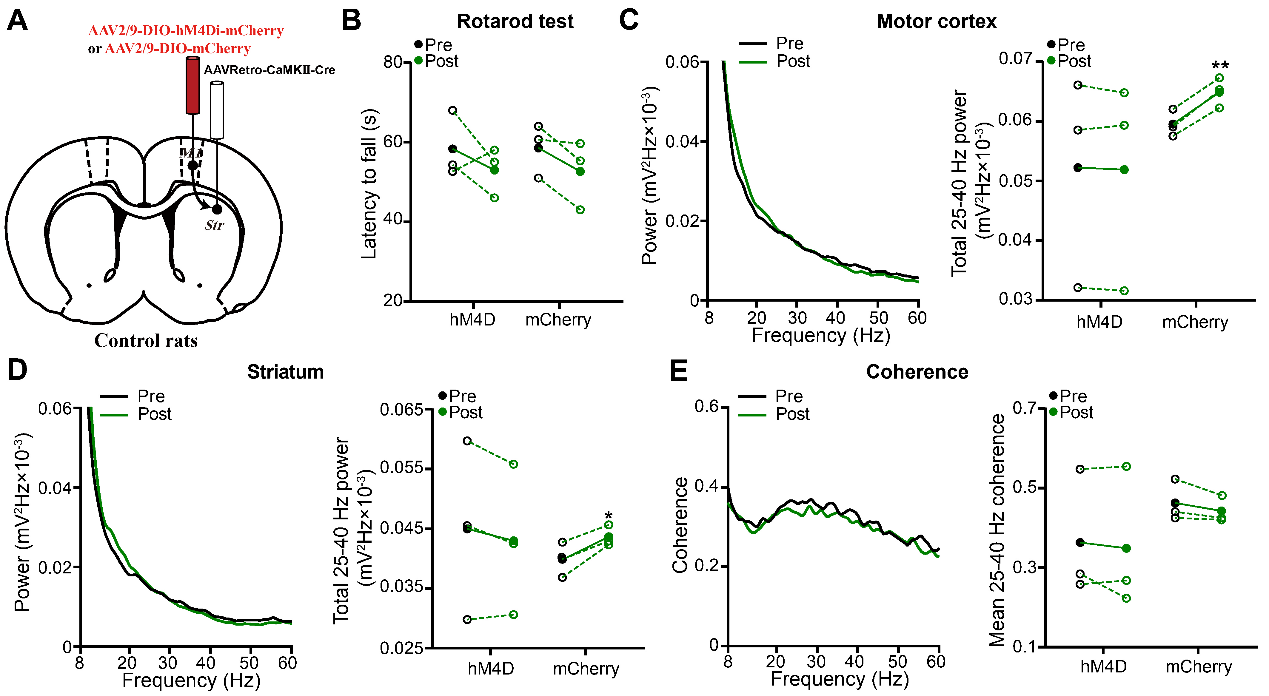


**Figure S5. Effects of corticostriatal glutamatergic projection inactivation in control rats.**  (A) Schematic illustration depicting the viral vector targeting approach. (B) Changes in the latency to fall in the rotarod test for the hM4D-CNO-treated and mCherry-CNO-treated control rats (CNO, i.p, 10 mg/kg). (Paired two-tailed t-tests; hM4D, *t*(2) = 0.970, *P* = 0.435; n = 3). (C) Left: Linear graph depicting the changes in the average power spectra in the motor cortex of hM4D-CNO-treated control rats before and after CNO administration (10 mg/kg, i.p.). Right: Changes in total high-beta (25-40 Hz) power in the motor cortex of hM4D-CNO- and mCherry-CNO-treated control rats before and after CNO administration (10 mg/kg, i.p.). (Paired two-tailed t-tests; hM4D, *t*(2) = 0.565, *P* = 0.629; n = 3). (D) Left: Linear graph depicting the changes in the average power spectra in the striatum of hM4D-CNO-treated rats before and after CNO administration (10 mg/kg, i.p.). Right: Changes in total high beta (25-40 Hz) power in the striatum of hM4D-CNO- and mCherry-CNO-treated rats before and after CNO administration (10 mg/kg, i.p.). (Paired two-tailed t-tests; hM4D, *t*(2) = 1.384, *P* = 0.301; n = 3). (E) Left: Linear graph depicting the change in corticostriatal high-beta coherence in hM4D-CNO-treated rats before and after CNO administration (10 mg/kg, i.p.). Right: Changes in the mean corticostriatal high-beta coherence of the hM4D-CNO- and mCherry-CNO- treated rats before and after CNO administration (10 mg/kg, i.p.). (Paired two-tailed t-tests; hM4D, *t*(2) = 0.638, *P* = 0.589; n = 3). The data are presented as the mean ± SEM. **p < 0.01, *p < 0.05 vs. preinjection.
